# Association Between 24-Hour Movement Behavior and Cognitive Function in Brazilian Middle-Aged and Older Adults: Findings From the ELSA-Brasil

**DOI:** 10.1093/geroni/igad030

**Published:** 2023-04-26

**Authors:** Natan Feter, Danilo de Paula, Rodrigo Citton P dos Reis, Sheila Maria Alvim Matos, Sandhi Maria Barreto, Bruce Bartholow Duncan, Maria Inês Schmidt

**Affiliations:** Post Graduate Program in Epidemiology, Federal University of Rio Grande do Sul, Porto Alegre, Rio Grande do Sul, Brazil; Post Graduate Program in Epidemiology, Federal University of Rio Grande do Sul, Porto Alegre, Rio Grande do Sul, Brazil; Post Graduate Program in Epidemiology, Federal University of Rio Grande do Sul, Porto Alegre, Rio Grande do Sul, Brazil; Statistics Department, Universidade Federal do Rio Grande do Sul, Porto Alegre, Rio Grande do Sul, Brazil; Post Graduate Program in Collective Health, Federal University of Bahia, Salvador, Bahia, Brazil; Faculdade de Medicina & Hospital das Clinicas/EBSERH, Universidade Federal de Minas Gerais, Brazil; Post Graduate Program in Epidemiology and Hospital de Clínicas de Porto Alegre, Federal University of Rio Grande do Sul, Porto Alegre, Rio Grande do Sul, Brasil; Post Graduate Program in Epidemiology and Hospital de Clínicas de Porto Alegre, Federal University of Rio Grande do Sul, Porto Alegre, Rio Grande do Sul, Brasil

**Keywords:** Cognition, Compositional analysis, Physical activity, Sedentary behavior

## Abstract

**Background and Objectives:**

The relationship between 24-hr movement behavior and specific domains of cognitive function is unclear. The purpose of this study was to identify the joint association of daily time spent in light (light-intensity physical activity [LPA]) and moderate-to-vigorous physical activity (MVPA), sedentary behavior (SB), and sleep with cognitive function in middle-aged and older adults.

**Research Design and Methods:**

Cross-sectional data from Wave 3 (2017–2019) of the Brazilian Longitudinal Study of Adult Health were analyzed. The study included adults aged 41–84 years old. Physical activity was assessed using a waist-worn accelerometer. Cognitive function was examined using standardized tests to assess memory, language, and Trail-Making test. Global cognitive function score was calculated by averaging domain-specific scores. Compositional isotemporal substitution models were performed to identify the association between the reallocation of time spent in LPA, MVPA, sleep, and SB with cognitive function.

**Results:**

Participants (*n* = 8,608) were 55.9% female (mean age 58.9 [8.6] years). Reallocating time from SB to MVPA was associated with higher cognitive function: Reallocating 15 min to MVPA by reducing 5 min from each other behavior was associated with increased odds of better cognitive function in both insufficient (<7 hr/day; odds ratio [OR]: 0.64; 95% confidence interval [CI]: 0.54–0.77) and sufficient (≥7 hr/day; OR: 0.62; 95% CI: 0.58–0.67) sleep groups. Among those with insufficient sleep, reallocating time to MVPA and sleep from SB was associated with higher global cognitive performance.

**Discussion and Implications:**

Small reductions in SB and increments in MVPA were associated with higher cognitive function in middle-aged and older adults.

**Translational Significance:** Evidence linking physical activity, sedentary behavior, and sleep with cognitive function based on a large, multiethnic, and cognitively diverse population are scanty. Our findings demonstrate that the reallocation of small amounts of sedentary behavior with physical activity was associated with improved cognitive function. Adequate distribution of time spent in physical activity, sedentary behavior, and sleep is required to preserve cognitive function. Based on the high prevalence of dementia and physical inactivity in low- and middle-income countries, our findings provide meaningful evidence that promoting physical activity, even in small doses, is associated with better cognitive function.

## Background

The association of physical activity, sedentary behavior (SB), and sleep patterns with cognitive function in older adults is well documented in the literature. Substantial evidence supports the benefits of physical activity in reducing the risk of cognitive impairment and dementia (Fratiglioni et al., 2020). Spending excessive time in SB (e.g., sitting and watching television) has been independently associated with lower cognitive performance (Falck et al., 2017) and increased risk of dementia (Yan et al., 2020). In addition, short and long sleep duration (less than 7 and more than 8 hr a day, respectively) have been associated with poor cognitive function and increased risk of incident dementia (Huang et al., 2022; Lo et al., 2016; Yan et al., 2020). However, the literature integrating these behaviors from a 24-hr daily composition perspective is scant.

The 24-hr movement behaviors composition paradigm considers that any increase of time in one behavior will imply that time spent on at least one of the other behaviors will decrease (Migueles et al., 2022). For example, increasing some minutes of physical activity per day requires a reduction in time of SB, sleep, or both. Also, the effects on health outcomes such as cognitive function associated with that increase are dependent on the substituted behavior because sleep and SB might have different associations with the outcome of interest. However, most studies investigating the associations of physical activity, SB, and sleep with health outcomes consider each as a stand-alone behavior. Recent statistical approaches such as compositional data analysis consider overall time-use composition. This methodology treats the behaviors as components of a total and deals with them in terms of proportions. Through this approach, it can be used to investigate the association of reallocations of time between different behaviors (Dumuid et al., 2019; Migueles et al., 2022).

Previous cross-sectional studies have used compositional data analysis to investigate the association between the 24-hr movement behavior with cardiometabolic biomarkers (Chastin et al., 2015; McGregor et al., 2019) and mortality (McGregor et al., 2021). However, studies examining the association between objectively measured physical activity, SB, and sleep with cognitive function are scarce (Mellow et al., 2022). Wei et al. (2021) reported that replacing 30 min/day of self-reported SB with self-reported moderate-to-vigorous physical activity (MVPA) was associated with improved cognitive function in adults. In another study, reallocating time to MVPA was associated with enhanced cognitive function in 82 middle-to-older adults, with a more prominent association among those with at least one apolipoprotein ɛ4 allele (Dumuid et al., 2022). However, the relationship between the 24-hr movement behavior and specific domains of cognitive function is still unknown. Also, the relatively small sample sizes limit the generalizability of the findings. Finally, most studies have suggested replacing at least 30 min of one behavior with another (Mellow et al., 2022). The number of barriers to lifestyle change and the high prevalence of physical inactivity in the older population is large. Thus, it is essential to examine the differences in cognitive function associated with small alterations, such as 15 min, in the distribution of movement behavior. To this end, we investigated the association of reallocating time between different behaviors with cognitive function in middle-aged and older adults in Brazil. We hypothesized that replacing short periods of SB with light-intensity physical activity (LPA) and MVPA would be associated with improved global and domain-specific cognitive function. In addition, we expected a nonlinear association between sleep duration and cognitive function.

## Method

### Participants

The Brazilian Longitudinal Study of Adult Health (*Estudo Longitudinal de Saúde do Adulto*, ELSA-Brasil) is an ongoing, multicenter cohort study investigating risk factors and determinants of chronic diseases in a free-living Brazilian population (Aquino et al., 2012). Baseline data were collected from 2008 to 2010 from 15,105 active and retired employees (aged 35–74 years) from public universities and research institutions in the capital of six Brazilian states (RS, SP, ES, RJ, MG, and BA). Trained and certified research assistants collected data via face-to-face interviews and clinical tests in the research centers (Aquino et al., 2012). The study has approval from the local research ethics committees. Participants signed consent forms for participation in all waves. To date, the study followed participants in two additional waves (2012–2014 and 2017–2019). Objectively assessed physical activity was first performed in the last of these (2017–2019).

### Exposure Variables

Participants were asked to continuously wear a triaxial accelerometer (ActiGraph wGT3X-BT, ActiGraph, Pensacola, FL) on the right side of the waist for 7 days, including asleep periods. The accelerometer captured and stored accelerations between −8 and 8 *g* (1 *g* = 9.8 m/s^2^) at a sampling frequency of 30 Hz. Participants also filled out a diary reporting the times they went to bed and woke up. Nonwear time was defined as blocks of 15 min with a standard deviation (*SD*) of acceleration ≤13 mg in a 60-min period (van Hees et al., 2013). Participants with at least 4 valid days of use (wear time ≥16 hr/day), with at least one on a weekend, were included in the data analysis (Trost et al., 2005). Time spent in SB, LPA, and MVPA was determined considering only the wake time based on valid cutoffs of acceleration for older adults, as follows (Sanders et al., 2019): SB: <19 mg (mili-g = 9.8 m/s^2^ × 10^−3^); LPA: 19–69 mg; MVPA: >69 mg. Sleep duration was assessed using the sleep diary. The adopted acceleration cutoffs were validated against direct observation of activities and reached high specificity for SB (97%) and high sensitivity for MVPA (94%; Sanders et al., 2019).

### Outcome Variables

Cognitive function was assessed using six standardized tests for the Brazilian, Portuguese-speaking population. Testing was applied in a quiet room by trained interviewers using standardized protocols, recorded, and reviewed for quality control (Bertola et al., 2021; Passos et al., 2014).

Memory and language were examined using a validated and adapted version of the Consortium to Establish a Registry for Alzheimer’s disease (CERAD) for a Brazilian, Portuguese-speaking population (Bertolucci et al., 2001). The memory test score represents the total number of correct words summing the learning, recall, and word recognition tests. Therefore, memory scores ranged from 0 to 50 and higher scores indicate better declarative memory (Morris et al., 1989). The language semantic test asked participants to name as many animals as possible, and the phonemic test asked them to say as many words starting with the letter F as they could in 1 min. Scores from semantic and phonemic language tests were summed, thus a higher number of recorded words indicates the better language (Morris et al., 1989). The Trail-Making test—Part B examined attention, concentration, psychomotor speed, visuomotor scanning, and mental flexibility (Lezak et al., 2004). Participants were instructed to connect 24 randomly placed circles in ascending order, alternating between numbers and letters, in the shortest time possible without lifting the pen from the paper. The score was the time (in seconds) spent completing the test. Scores were multiplied by −1, so that higher scores indicated better performance. In addition, because of their highly skewed distribution, scores were log-transformed.

Scores from each domain (memory, language, and trail B) were standardized in *Z* scores based on a mean and *SD* of 0 and 1. Values were adjusted for age (mean = 59.6 years), sex, and education. Next, a global cognitive function score was derived by averaging the domain-specific *Z* scores. The global score was also standardized on a scale with zero mean and an *SD* of 1 (Heffernan et al., 2019; Rojer et al., 2021; Singh et al., 2014). In addition, global cognitive function scores were categorized into deciles, with participants in the lowest (first) decile being classified as having a poor cognitive function.

### Covariates

Covariates were also obtained in the study research centers via face-to-face interviews and clinical assessments. Sociodemographic characteristics included age, sex, education, and self-declared race/skin color (white, brown (*pardo*), black, yellow [Asian], and indigenous). Health behaviors included smoking (nonsmoker, ex-smoker, and smoker) and excessive alcohol consumption (≥210 g/week for men and ≥140 g/week for women; Chor et al., 2013). The presence of common mental disorders was defined as a score of ≥12 on the Clinical Interview Schedule-Revised (CIS-R; Lewis et al., 1992; Nunes et al., 2016). Weight and height were obtained while fasting to calculate the body mass index. Diabetes was defined as HbA1c ≥6.5% (48 mmol/mol), fasting glycemia ≥126 mg/dL (7.0 mmol/L), or a 2-hr value during a 75 g oral glucose tolerance test ≥200 mg/dL (11.1 mmol/L), by the use of glucose-lowering medication, or by a self-reported medical diagnosis of diabetes (Schmidt et al., 2014). Blood pressure was measured using a validated oscillometric device (Omron HEM 705CPINT) on the right arm after a 5-min rest in a sitting position in a quiet room, with a controlled temperature (20–24°C). Three measurements were taken at 1-min intervals, and the mean of the last two measurements of SBP and DBP was used (Mill et al., 2013). Individuals with SBP ≥ 140 mm Hg, DBP ≥ 90 mm Hg. or use of antihypertensive medication were classified as having hypertension (de Menezes et al., 2021).

### Statistical Analysis

Analyses were conducted using STATA v.14.2 (Stata Corp, College Station, TX) and R Version 4.2.1 (The R Foundation for Statistical Computing, Vienna, Austria; R Core Team, 2021). Compositional analyses were performed using the “epicoda” package (Walmsley et al., 2021). The categorical variables were expressed as frequencies and percentages. Continuous variables were described as the mean and *SD* or the median and interquartile range (IQR), depending on the normality of the data distribution, assessed using histograms and the asymmetry coefficients skewness.

Models were additionally adjusted for study center, race/skin color, smoking, excessive alcohol consumption, body mass index, and common mental disorder. Based on a previous demonstration that the association between sleep time and cognitive function is not linear, with a sleep duration of 7 hr per night being optimal (Richards et al., 2017), we used linear and quadratic regression models to examine the linear and nonlinear associations of sleep duration with global cognitive function. If a significant quadratic association was found, restricted cubic spline models were fitted to describe the shape of the dose–response curves of total sleep duration with cognition, using knots at the 10th, 50th, and 90th percentiles of sleep duration (Harrell, 2001). Logistic regression models were used to quantify the association between the time reallocation of individual behaviors and poor cognitive function.

### Compositional Analyses

A time-use composition of the time in SB, LPA, MVPA, and sleep was formed using compositional data analysis. Previous studies have extensively described the mathematical basis of compositional analysis (Chastin et al., 2015, 2021). Briefly, each part of the composition is expressed relative to the other components by creating isometric log-ratio variables. Therefore, participants’ time spent in sleep, SB, LPA, and MVPA has transformed into three isometric log-ratio (ilr) coordinates (Chastin et al., 2021). The proportion of time spent in the different behaviors was reported as arithmetic and compositional means. The compositional mean, or center, is the vector of geometric means of its parts, rescaled, to sum up to 24 hr, and is coherent with the interdependent nature of compositional data (Chastin et al., 2015; Dumuid et al., 2020). Recorded days can be of different duration. To standardize this duration to 24 hr, we first verified that no participant had an average day’s duration shorter than 23 or longer than 25 hr. We then multiplied, for each day, the proportion of time spent in each of the four behaviors by 24, so that all days for all participants would be composed of 24 hr.

We present pairwise time reallocation plots, which show the theoretical effect of reallocating time from one behavior to another. In addition, we estimated the predicted *Z*-score differences and odds ratios [ORs] of poor cognitive function associated with time reallocations between behaviors (e.g., reallocating 15 min/day to MVPA from all other behaviors proportionally). All results are relative to the average behavior composition of our sample and should be interpreted as the outcome associated with reallocating time between behaviors for the average individual in our sample.

### Sensitivity Analyses

We performed sensitivity analyses considering daily mean acceleration categorized in deciles, with the first decile representing the lowest daily mean acceleration. In addition, we reanalyzed the results adjusting additionally for the presence at baseline of diabetes and hypertension, the study research center, and daily mean acceleration.

## Results

### Descriptive Results

**Figure 1.**
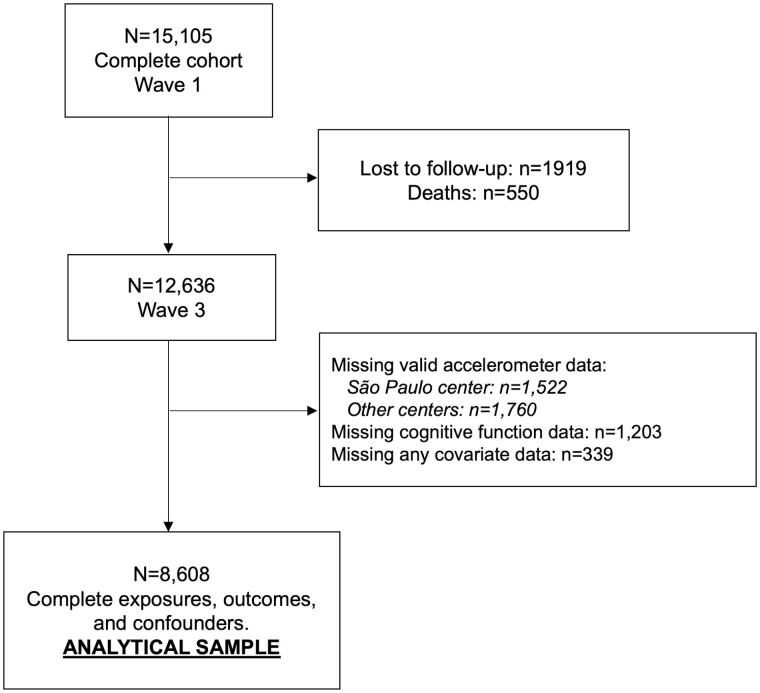
Flowchart of study participation.

After excluding participants with missing data, our analytic sample was composed of 8,608 middle-aged and older adults (Figure 1). Most were female (55.9%), White (55.2%), and had at least a university degree (57.9%). Their average time spent in SB, LPA, MVPA, and sleep was 12.3 hr/day, 3.4 hr/day, 48.2 min/day, and 7.4 hr/day, respectively (Table 1). Most (*n* = 5,505, 64.5%) slept more than 7 hr/day. In addition, we observed a positive dose–response association between age and prevalence of cognitive impairment, with a higher proportion of cases in those aged 70 or older at baseline (30–39: 0.3%; 40–49: 3.3%; 50–59: 12.8%; 60–69: 20.0%; 70 or older: 35.1%).

**Table 1.** Sample Characteristics of the Included Participants (*N* = 8,608)

Characteristic	Mean (*SD*)	*N* (%)	Median (IQR)
Age, years	58.9 (8.6)		
Sex			
Male	3,792 (44.1)		
Female	4,816 (55.9)		
Race or race/ethnicity			
Black		1,239 (14.4)	
Mixed race (*pardo*)		2,325 (27.0)	
White		4,755 (55.2)	
Asian or Indigenous		289 (3.4)	
Highest educational achievement			
Less than elementary school		237 (2.7)	
Elementary school		416 (4.8)	
High school		2,972 (34.5)	
University degree or higher		4,983 (57.9)	
Smoking			
Never smoker		5,294 (61.5)	
Former smoker		2,589 (30.1)	
Current smoker		725 (8.4)	
Excessive alcohol drinking, yes^a^		1,478 (17.2)	
Sleep, hr/night			
Insufficient (<7)		3,056 (35.5)	
Sufficient (≥7)		5,552 (64.5)	
Body mass index			
Overweight		3,674 (42.6)	
Obese		2,372 (27.6)	
Diabetes, yes		2,067 (24.0)	
Hypertension, yes		3,752 (43.9)	
Common mental disorder, yes^b^		2,086 (24.2)	
Memory, number words recalled^c^	37.6 (5.9)		
Language, number words recalled^d^	31.7 (8.0)		
Trail-Making Test (part B), seconds^e^			98 (73–139)
Time spent			
In sedentary behavior^f^ (hr/day)	12.3 (1.7)		
In light-intensity physical activity^g^ (hr/day)	3.4 (1.1)		
In moderate-to-vigorous physical activity^h^ (min/day)	47.8 (25.5)		
In sleep^i^ (hr/day)	7.4 (1.2)		

*Notes*: IQR = interquartile range; *SD* = standard deviation.

^a^ ≥210 g of alcohol/week for men and ≥140 g of alcohol/week for women.

^b^ Score ≥12 in the *Clinical Interview Schedule-Revised* (CIS-R).

^c^ Range from 0 to 50.

^d^ Range from 0 to ∞.

^e^ Time to complete the task.

^f^ Acceleration ≤15 m*g.*

^g^ Acceleration 16–69 m*g.*

^h^ Acceleration >69 m*g.*

^i^ Reported in sleep diary.

As shown in Supplementary Figure 1, we found a quadratic association between sleep duration and cognitive function in adjusted restricted cubic spline plots, supporting our use of a daily sleep duration of 7 hr as optimal for cognition. Therefore, analyses henceforward were conducted separately to those having insufficient (<7 hrs/day) and sufficient (≥7 hr/day) sleep duration times. Supplementary Table 1 and Supplementary Figures 2 and 3 illustrate the arithmetic and compositional mean time spent in each movement behavior. Participants with insufficient sleep spent more of their wake time in SB than those with sufficient sleep (55.6% [55.3%, 55.8%] and 49.2% [49.0%, 49.4%], respectively), with no tangible differences between the time spent in MVPA and LPA.

### Proportional Substitutions of Behaviors

**Figure 2.**
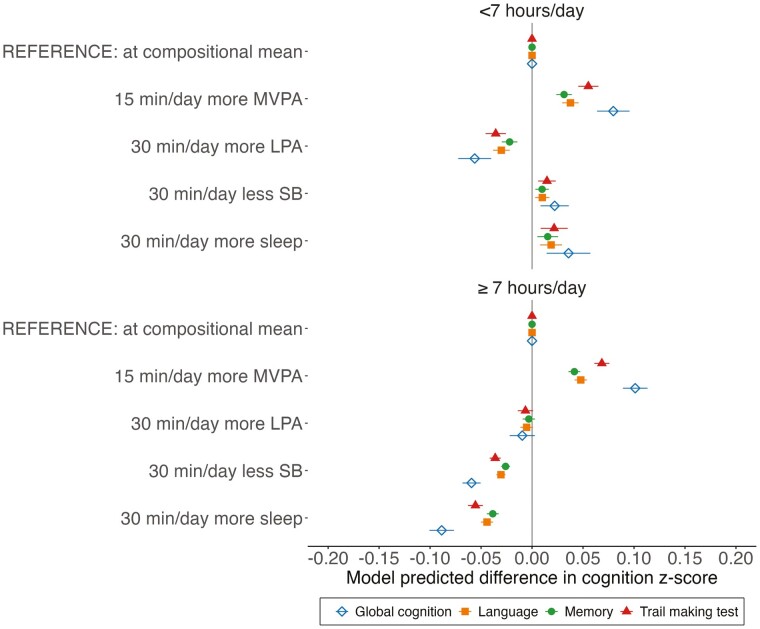
Predicted age, sex, and education-standardized global and domain-specific cognitive function associated with reallocating time to named behavior, from all other behaviors proportionally in participants with (A) insufficient (<7 hr/day) and (B) sufficient (≥ 7 hr/day) sleep duration. LPA = light-intensity physical activity; MVPA = moderate-to-vigorous physical activity; SB = sedentary behavior. Mean behavior composition: insufficient sleep: 6.3 hr/day sleep, 13.5 hr/day SB, 3.5 hr/day LPA, 45 min/day MVPA; sufficient sleep: 8.2 hr/day sleep, 11.9 hr/day SB, 3.2 hr/day LPA, 38 min/day MVPA. Models were adjusted for study center, race/ethnicity, body mass index, smoking, excessive alcoholic consumption, and common mental disorders.

As illustrated in Figure 2, reallocating 15 min to MVPA proportionally was associated with improved global and domain-specific cognitive function regardless of sleep duration. The same association was observed in the odds of poor cognitive function: Increasing 15 min of MVPA by reducing 5 min from each additional behavior was associated with reduced odds of poor cognitive function in both insufficient (OR: 0.64; 95% confidence interval [CI]: 0.54, 0.77) and sufficient (OR: 0.62; 95% CI: 0.58, 0.67) sleep groups (Supplementary Figure 4). Decreasing 30 min of SB proportionally was associated with worse global and domain-specific cognitive function among those with sufficient sleep; however, a discrete inverse association was observed in those with insufficient sleep (Figure 2). The same pattern of associations of SB was observed when increasing 30 min of sleep, with an even stronger association in those with sufficient sleep. Adding 30 min of LPA proportionally was associated with lower global and domain-specific cognitive function scores in the group with insufficient sleep; no associations were observed in those with sufficient sleep.

### Substitution of Sedentary Behavior With Specific Activities

**Figure 3.**
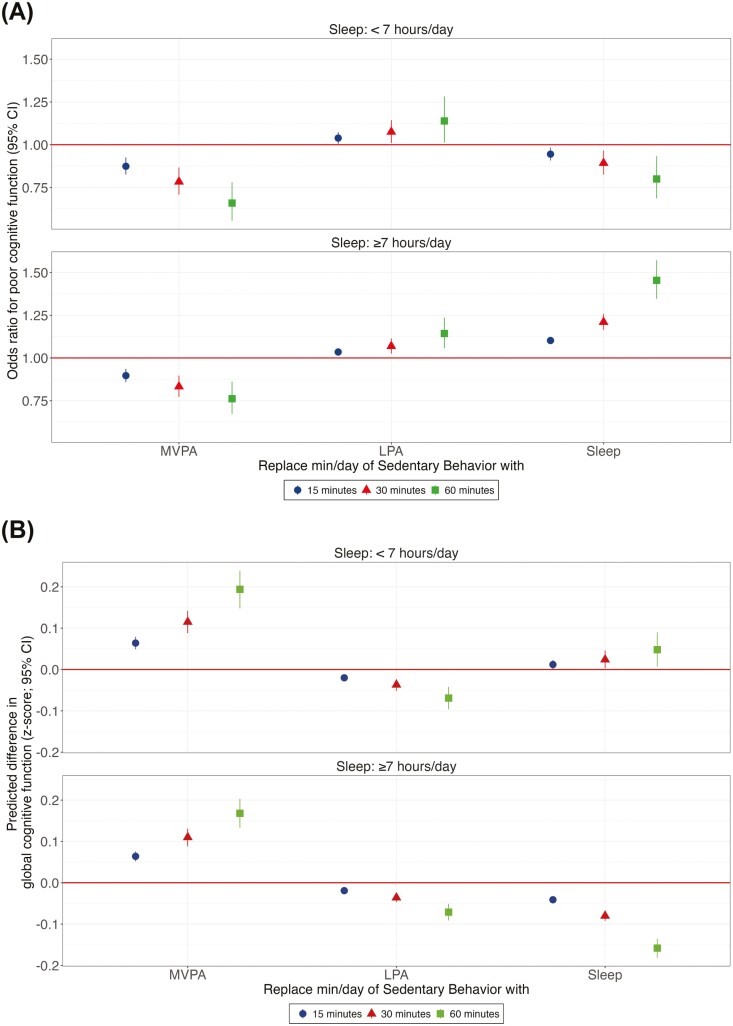
Association between isotemporal substitution of 15, 30, and 60 min in SB with other behaviors (MVPA, LPA, and sleep) and age, sex, and education-standardized global cognitive function scores (A) and poor cognitive function (B) in Brazilian adults stratified by sleep duration. SB = sedentary behavior; LPA = light-intensity physical activity; MVPA = moderate-to-vigorous physical activity. Cognitive impairment was defined as the first (lowest) decile on global cognitive score. Models were adjusted for study center, race/ethnicity, body mass index, smoking, excessive alcoholic consumption, and common mental disorders.

As illustrated in Figure 3A, reallocating time from SB to MVPA was associated with improved global cognitive function regardless of sleep duration. A dose–response association could be observed, with a larger magnitude in those with insufficient sleep. The same association was observed in the odds of poor cognitive function (Figure 3B). Predicted differences in domain-specific cognitive function resulting from the reallocation of time from SB to others are described in detail in Supplementary Table 2.

Substituting time in SB with LPA was associated with lower cognitive function scores (Figure 3A) and higher odds of poor cognitive function (Figure 3B and Supplementary Table 3) in both sleep groups.

In those with insufficient sleep, increasing sleep duration at the cost of SB was associated with higher global cognition (Figure 3A) and lower odds of poor cognitive function (Figure 3B). Alternatively, in those with sufficient sleep, exchanging SB for sleep was associated with lower cognitive function scores and higher odds of poor cognitive function, with a stronger association than the benefit of the insufficient sleep group.

**Figure 4.**
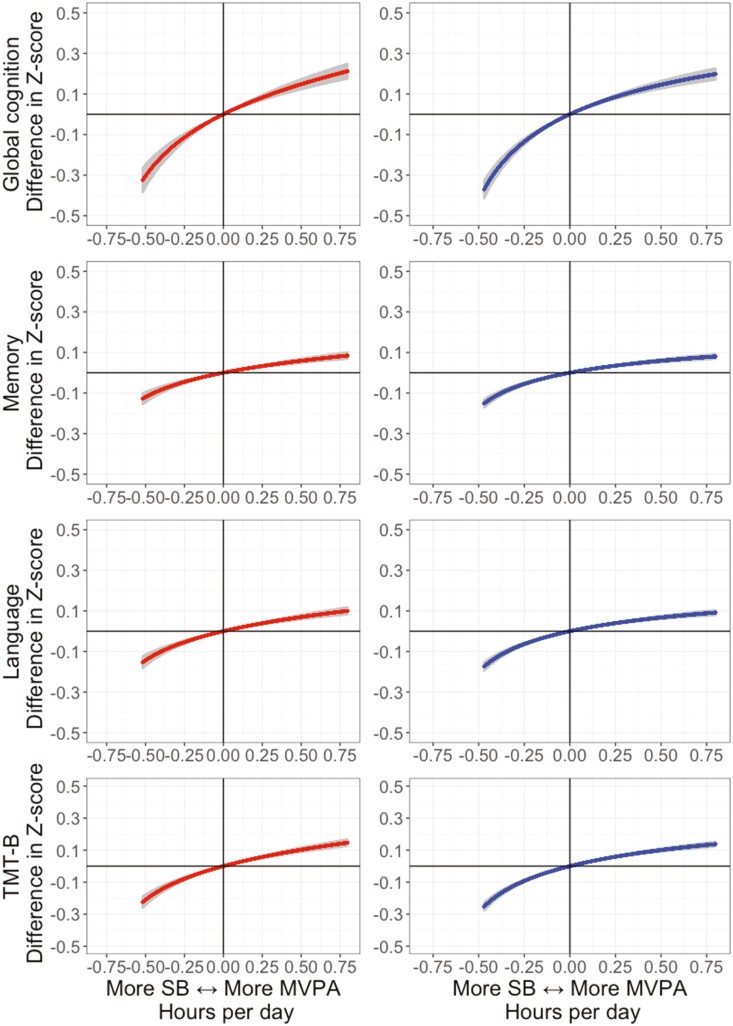
Predicted age, sex, and education-standardized global and domain-specific cognitive function as result of reallocating time between SB and MVPA in compositional analysis. LPA = light-intensity physical activity; MVPA = moderate-to-vigorous physical activity; SB = sedentary behavior; TMT-B = Trail-Making test—part B. Insufficient (<7 hr/day) and sufficient (≥7 hr/day) sleep hours are represented in left-sided column and right-sided column, respectively. Models were adjusted for study center, race/ethnicity, body mass index, smoking, excessive alcoholic consumption, and common mental disorders. Compositional references: sleep: 6.5 hr/day and LPA: 3.5 hr/day (insufficient sleep); sleep: 8.2 hr/day and LPA: 3.2 hr/day (sufficient sleep).

Figure 4 and Supplementary Figures 5 and 6 show the changes in cognitive function scores and the odds of poor cognitive function associated with the bidirectional substitutions of time between MVPA and SB. Increasing 30 min of SB at the cost of MVPA was associated with lower cognitive function scores in both insufficient (−0.31; 95% CI: −0.37, −0.25 *Z* score) and sufficient (−0.43; 95% CI: −0.49, −0.38 *Z* score) sleep groups. Increasing 30 min of MVPA by reducing SB was associated with higher cognitive function scores in both insufficient (0.15; 95% CI: 0.12, 0.17 *Z* score) and sufficient (0.14; 95% CI: 0.12, 0.17 *Z* score) sleep groups.

A similar pattern was observed when comparing the odds of poor cognitive function. Reallocating 30 min from SB to MVPA was associated with lower odds in both insufficient (OR 0.74; 95% CI: 0.67; 0.82) and sufficient (OR 0.78; 95% CI: 0.73; 0.84) sleep groups. Increasing 30 min of MVPA by reducing SB was associated with higher odds in insufficient (OR 1.84; 95% CI: 1.49; 2.28) and sufficient (OR 2.16; 95% CI: 1.79; 2.61) sleep groups.

For an average individual, reallocating time from LPA or sleep to MVPA was associated with higher global cognitive performance (Supplementary Figure 7). In those with insufficient sleep duration, reallocating time from LPA to sleep was also associated with improved cognitive function, and reallocating time from sleep to SB and from SB to LPA was associated with small decreases in global cognitive function. In participants with sleep duration ≥7 hr/day, reallocating time from sleep to LPA and SB was associated with improved global cognitive function (Supplementary Figure 8).

Decreasing sleep duration by adding time in MVPA was not associated with better memory and language function (Supplementary Figures 9–14). Replacing MVPA with sleep was associated with worse cognitive scores. Adding time in MVPA by reducing SB, sleep, and LPA was associated with improved TMT-B performance. No predicted difference in cognitive function was observed in other tested analyses.

### Sensitivity Analyses

When diabetes and hypertension were added, in insufficient sleepers, replacing time in SB with sleep showed lower odds of poor global and domain-specific cognitive function. However, displacing time in SB with MVPA was associated with lower odds only of TMT-specific poor cognitive function (Supplementary Tables 4 and5, Supplementary Figures 15 and16).

We also stratified the sample into subgroups defined by acceleration deciles (Supplementary Table 6, Supplementary Figure 17). In the lowest acceleration decile, the association between replacing SB with MVPA was more pronounced than that seen in the primary analysis, although the association between SB and LPA was not confirmed.

## Discussion

Our findings show that daily reallocating small amounts of SB with MVPA was associated with higher cognitive function in middle-aged and older adults. Among those with sufficient sleep (>7 hr/day), reallocating time in sleep with MVPA was also associated with higher cognitive function. Among those with insufficient sleep duration, increasing MVPA and sleep duration was associated with higher cognitive function; however, the increased time spent in these activities must be reallocated from either SB or LPA. In addition, most of our findings for domain-specific cognitive function scores mirrored the findings for the global cognitive function score. There was a clear dose–response association between reallocating time from SB, LPA, and sleep to MVPA and global and domain-specific cognitive function. Reallocating 15 min from SB to MVPA was associated with higher global cognitive function in both insufficient and sufficient sleepers. This improvement represented increased odds of better cognitive function by up to 13% (Supplementary Table 3).

A systematic review of the association between objectively measured physical activity and cognitive function(Rojer et al., 2021) showed that higher MVPA was associated with better cognitive function in older adults with an average effect size (standardized β) of 0.16 (IQR: 0.069, 0.285). For comparison, a cognitive decline of −0.27 *SD* (95%CI: −0.29, −0.24) during a 10-year follow-up was associated with an increased risk of dementia in older adult participants of the Whitehall II study (Kaffashian et al., 2013). A dose-dependent association between time spent in MVPA and executive function and the risk of cognitive impairment was also found by other studies (Zhu et al., 2017), with a yearly decline in executive function (−0.01 *Z* score) over a 3-year follow-up, similar to that seen in a previous study (Beker et al., 2021). However, participants who spent ≥3% of accelerometer wear time with MVPA at baseline improved this cognitive domain (+0.04 *Z* score) and reduced the risk of cognitive impairment (OR: 0.64; 95% CI: 0.48, 0.84) in the same period. The predicted difference in global cognitive function due to the reallocation of 15 min from SB to MVPA may reflect in slower cognitive decline. However, this hypothesis must be investigated by future longitudinal studies.

The guideline for daily movement behavior implies the existence of an optimal combination of movement behaviors (e.g., high LPA and MVPA, low SB, and adequate sleep; Rollo et al., 2020). For example, higher time spent in MVPA has been associated with greater brain volume, circulating levels of brain-derived neurotrophic factor, and synaptic plasticity (Hillman et al., 2008; Neves et al., 2022), ultimately resulting in improved cognitive function and reduced risk of dementia (Petermann-Rocha et al., 2021; Rojer et al., 2021). However, our results suggest that tailored physical activity promotion strategies should consider the distribution of movement behaviors in the 24 hr to achieve optimal results. In the present study, an average participant with insufficient sleep duration should preserve or increase MVPA; however, he or she should also increase sleep duration. The latter can be achieved by reducing either SB or LPA, as decreasing MVPA to improve sleep showed a deleterious association with cognitive function. Similarly, they suggest that participants with ≥7 hr of sleep per day should also preserve or increase MVPA and would benefit additionally with a reduction in sleep duration toward the recommended 7 hr per night. However, replacing excessive sleep time with MVPA rather than with SB was associated with a higher predicted improvement in cognitive function.

Furthermore, the predicted worsening in cognitive function from reallocating time in MVPA with SB or sleep was, on average, 3.5 times greater in magnitude, than the positive increment associated with more MVPA (Figure 4). MVPA and SB have inversed associations with health outcomes. However, considering the whole of 24-hr movement behavior, one can be both highly physically active and highly sedentary. For example, the analyzed sample had an average volume of physical activity of 296.1 min of MVPA per week, 2 times larger than the minimum recommended by the 2020 WHO guidelines of physical activity and SB (Bull et al., 2020). However, the same subjects spend at least 50% of the 24 hr with sedentary activities. Even for this highly active sample, reallocating short amounts of MVPA with SB was deleterious for cognitive function regardless of the sleep status. Previous evidence suggests that for adults with 8+ hr of accumulated SB per day, at least 1 hr of MVPA per day is necessary to mitigate the risk of all-cause mortality related to SB (Ekelund et al., 2016). As previously cited, physical activity and SB have been consistently associated with cognitive function in older adults (Ekelund et al., 2016; Mellow et al., 2022). Our findings indicate that the association may not be symmetrical, with more SB being more deleterious for cognitive function than an equivalent reduction in SB is beneficial in these highly active middle-aged and older adults.

Moreover, previous corroborating findings showed a quadratic, nonlinear association between sleep and cognitive function (Mellow et al., 2022). Wei et al. (2021) and Kimura et al. (2019) showed that in older adults with sleep duration >7 and ≥8 hr/day, respectively, total sleep time had a negative association with cognitive function. On the other hand, a positive association was observed in those who engaged in <7 hr/day (Wei et al., 2021). Sleep has been confirmed as a critical lifestyle behavior for several health outcomes, including cognitive function and risk of dementia (Huang et al., 2022; Lo et al., 2016). Considering the 24-hr movement guidelines, excessive sleep duration will inevitably be associated with decreased time in either SB or physical activity. On the other hand, extra time awake resulting from insufficient sleep will increase time in either behavior. The literature provides substantial evidence for protection by physical activity, especially MVPA, and appropriate duration of sleep against cognitive impairment and dementia (Fratiglioni et al., 2020; Huang et al., 2022; Iso-Markku et al., 2022; Lo et al., 2016). Our data suggest that the right balance between SB, sleep, and physical activity should be prioritized. Attention to this balance may be especially relevant for women whose work–family conflict affects these behaviors (Patrão et al., 2017). Future public health policies may focus on promoting leisure time and commuting physical activity through improvement in walkability and increased access to shared space to physical activity practice aligned with health literacy strategies oriented by 24-hr movement guidelines.

In the present study, participants with insufficient sleep duration showed higher daily time in SB but no meaningful difference in the time spent in LPA or MVPA from those with greater sleep duration. Increased time in SB has been associated with better cognitive function in previous studies (Mellow et al., 2022; Wei et al., 2021). Greater SB may result from greater time pursuing cognitively demanding activities such as reading and solving puzzles which produce minimal acceleration but have been associated with improved cognitive function (Mellow et al., 2022; Sajeev et al., 2016). Objectively measured physical activity and SB provide reliable and precise information about one’s 24-hr movement pattern. However, it is not possible to precisely detail which specific physical and sedentary activities the participants were performing. This study’s sample comprised active and retired employees from public universities and research institutions. A multicohort study analyzing data from 107,896 workers in Europe, the United Kingdom, and the United States (Kivimäki et al., 2021) indicated that greater cognitively demanding activity at work was associated with a lower risk of all-cause dementia. The same research highlighted that stimulating working activities were also associated with reduced expression of proteins related to brain damage. These findings support the positive association we found for reallocating time from LPA to SB. Therefore, it is reasonable to hypothesize that some of the remarkable proportion of time spent in SB in this cohort may be spent in cognitively demanding activities, which may explain the observed positive association of greater SB with greater cognitive function among those with insufficient sleep. Future waves of the ELSA-Brasil study will investigate the association of different types of sedentary, cognitively demanding activities on cognitive function. This approach may provide an essential understanding of future SB guidelines for the older population (Mellow et al., 2022).

Our study has some limitations that need to be acknowledged. First, the cross-sectional design does not allow causal inference in reallocating time between behaviors and cognitive function. However, previous systematic reviews (Mellow et al., 2022; Rojer et al., 2021) highlighted the lack of studies investigating the 24-hr movement behavior on cognitive function in older adults, which is the gap this study aimed to reduce. Second, sleep was assessed by a self-reported diary, an indirect method, and is potentially subject to bias related to the subjectivity of the information provided. Third, we could not identify different SBs (e.g., cognitively demanding vs not demanding). Finally, accelerometer data were not available for all participants in one center (Figure 1). However, sensitivity analysis showed no difference in sociodemographic and clinical characteristics in participants according to the availability of accelerometer data (Supplementary Table 7).

Our study has also strengths that merit mention. ELSA-Brasil is a large cohort of middle-aged and older Brazilians with highly standardized sociodemographic, anthropometric, cognitive, and 7-day accelerometer measurements, which is rarely accomplished worldwide, particularly in low- and middle-income countries. To our knowledge, this is the first study to investigate the joint association of objectively measured physical activity, sedentary behavior, and sleep with cognitive function in a multiethnic population with diverse cognitive reserves of middle-aged and older adults living in Latin America.

## Conclusion

In conclusion, our findings support the “every step count” statement by the WHO guideline of physical activity and SB. Reallocating small doses of SB with MVPA was associated with higher cognitive function and lower odds of poor cognitive function in middle-aged and older adults. We provided new information on the importance of considering the 24-hr movement behavior guidelines in promoting physical activity to preserve and improve cognitive function.

## Supplementary Material

igad030_suppl_Supplementary_Materials

## Data Availability

The data that support the findings of this study are available from the corresponding author on request.

## Patient and Public Involvement

Patients were not involved in the study design or data analysis; however, participants of the ELSA study received individual reports of their clinical evaluations.
