# Supplementary material for: Association Between 24-Hour Movement Behavior and Cognitive Function in Brazilian Middle-Aged and Older Adults: Findings From the ELSA-Brasil
Source: Innov Aging. 2023 Apr 26;7(3):igad030. doi: 10.1093/geroni/igad030 (PMC10184510; doi:10.1093/geroni/igad030)
Supplement: igad030_suppl_Supplementary_Materials [file igad030_suppl_supplementary_materials.docx]

**Online** **Supplementary Material**

**Supplementary Figure 1.** Quadratic association of sleep duration (hours/day) with the global cognitive function in middle-aged and older adults. The solid blue line represents the estimated mean global cognitive function as Z-score and shaded area represents the 95% confidence intervals. The restricted cubic spline with Harrell’s knots (10th, 50th, and 90th percentile of sleep duration per day) was fitted with adjustment for age, sex, education, race/ethnicity, body mass index, smoking, excessive alcoholic consumption, and common mental disorders.

**
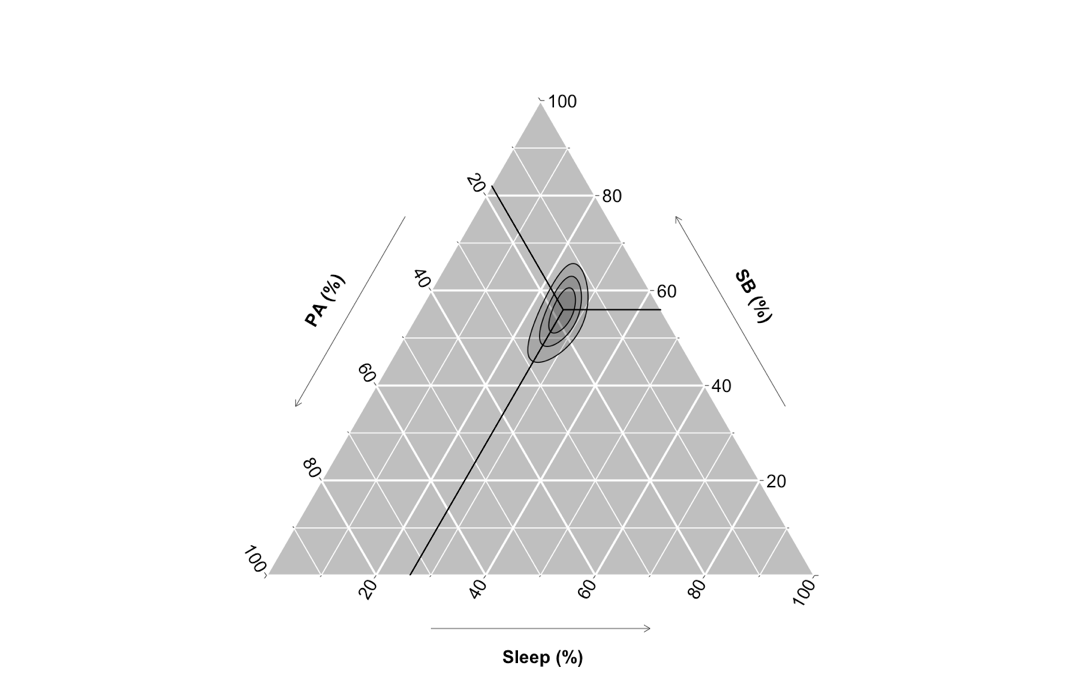

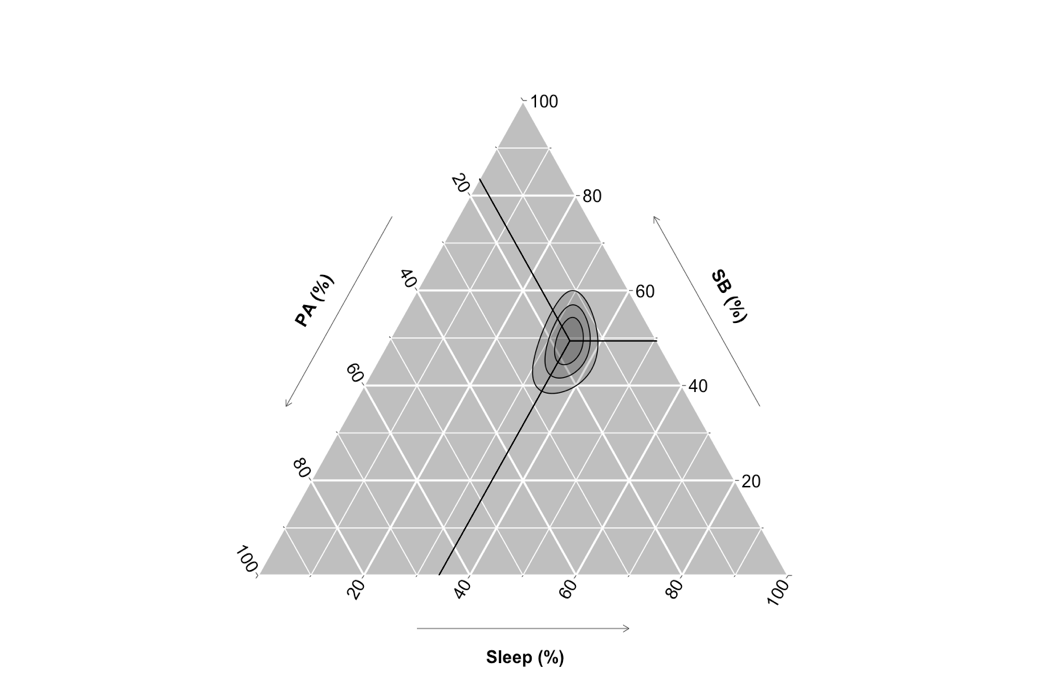
**

A

B

**Supplementary Figure 2.** Movement behaviors of participants with (A) insufficient and (B) sufficient sleep duration on a ternary plot, showing sleep, SB, and PA behaviors (PA: combines light and moderate-to-vigorous physical activity). The crosshair marks the compositional mean. Concentric rings represent the 25, 50% and 75% confidence regions for the data. The behavior composition at a point can be found by tracing out (parallel to the white lines and crosshair) from the point to the axes. PA: physical activity; SB: sedentary behavior

B

A

**Supplementary Figure 3.** Distribution of the 24-hour movement behaviors of participants with sleep duration (A) <7 hours/day and (B) ≥7 hours/day, showing sleep, SB, LPA, and MVPA stratified by sleep duration. LPA, light physical activity; MVPA, moderate-to-vigorous physical activity; SB: sedentary behavior.


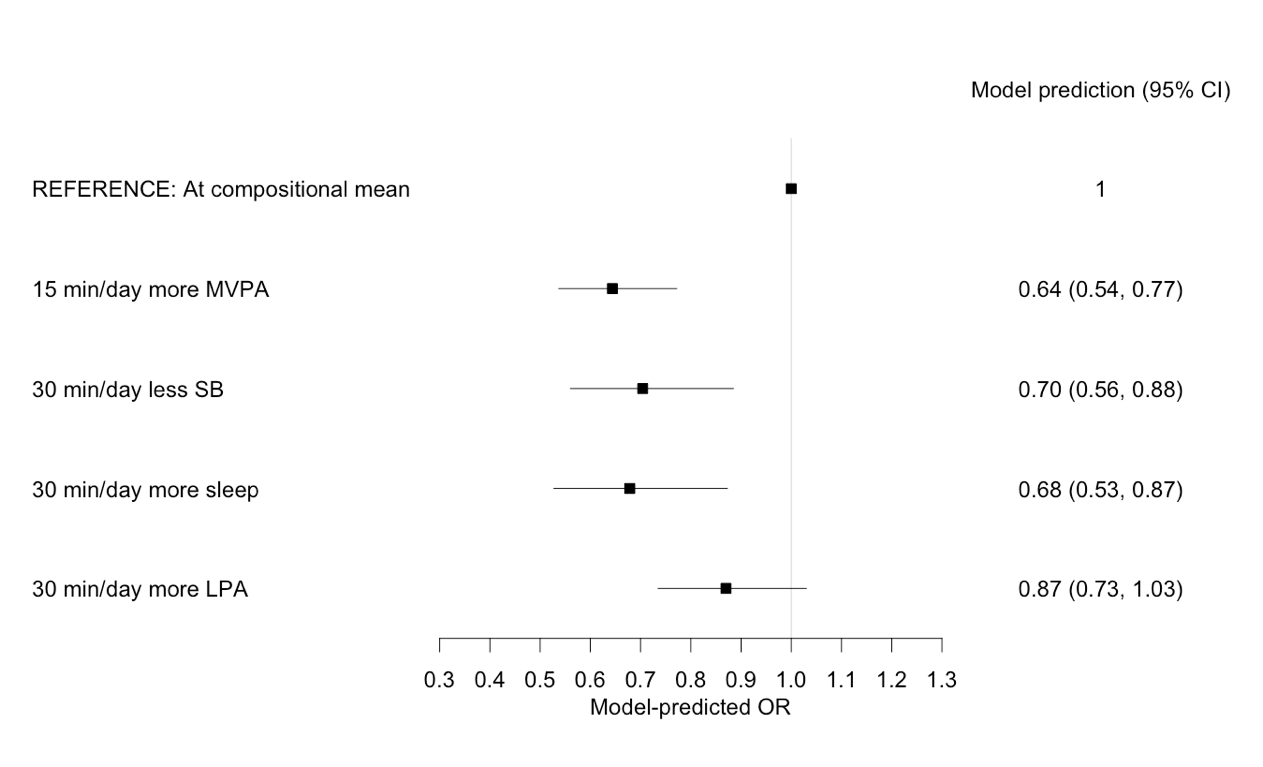


A

**
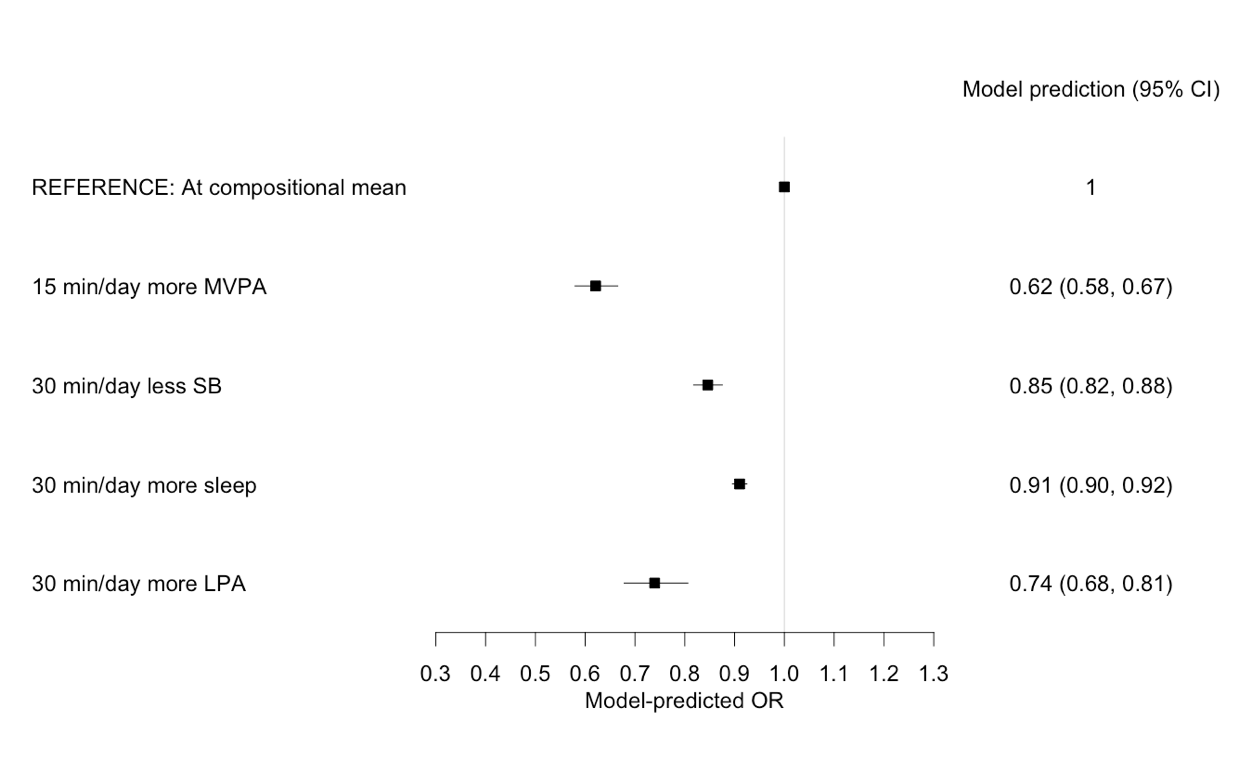
Supplementary Figure 4.** Predicted odds of poor cognitive function associated with reallocating time to named behavior, from all other behaviors proportionally in participants with (A) insufficient (< 7 hours/day) and (B) sufficient (≥ 7 hours/day) sleep duration. Mean behavior composition: insufficient sleep: 6.3 hours/day sleep, 13.5 hours/day SB, 3.5 hours/day LPA, 45min/day MVPA; sufficient sleep: 8.2 hours/day sleep, 11.9 hours/day SB, 3.2 hours/day LPA, 38 min/day MVPA. Models were adjusted for study center, race/ethnicity, body mass index, smoking, excessive alcoholic consumption, and common mental disorders. Poor cognitive function was defined as age, sex, and education-standardized scores in the first (lowest) decile of the global cognitive function. LPA: light physical activity; MVPA: moderate-to-vigorous physical activity. SB: sedentary behavior.

B


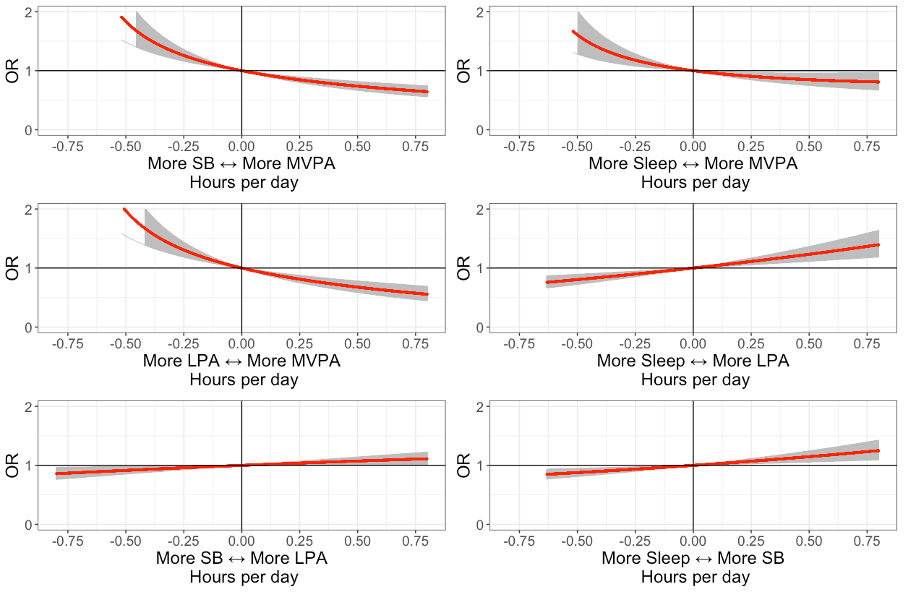


**Supplementary Figure 5.** Predicted odds ratio (95% confidence zone) of poor cognitive function as result of reallocating time between behaviors in participants with Insufficient (<7 hours/day) sleep hours. Models were adjusted for study center, race/ethnicity, body mass index, smoking, excessive alcoholic consumption, and common mental disorders. Poor cognitive function was defined as age, sex, and education-standardized scores in the first (lowest) decile of the global cognitive function. Compositional references: 6.3 hours/day sleep, 13.5 hours/day SB, 3.5 hours/day LPA, 45min/day MVPA. MVPA: moderate-to-vigorous physical activity. SB: sedentary behavior; LPA: light physical activity. OR: odds ratio.


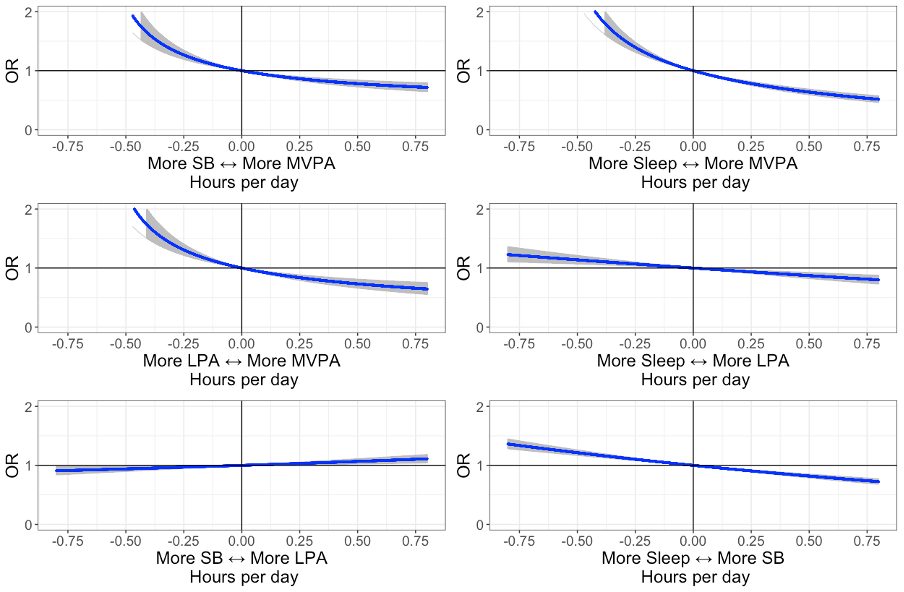


**Supplementary Figure 6.** Predicted odds ratio (95% confidence zone) of poor cognitive function as result of reallocating time between behaviors in participants with sufficient (≥7 hours/day) sleep hours. Models were adjusted for study center, race/ethnicity, body mass index, smoking, excessive alcoholic consumption, and common mental disorders. Poor cognitive function was defined as age, sex, and education-standardized scores in the first (lowest) decile of the global cognitive function. Compositional references: 8.2 hours/day sleep, 11.9 hours/day SB, 3.2 hours/day LPA, 38 min/day MVPA. MVPA: moderate-to-vigorous physical activity. SB: sedentary behavior; LPA: light physical activity. OR: odds ratio.


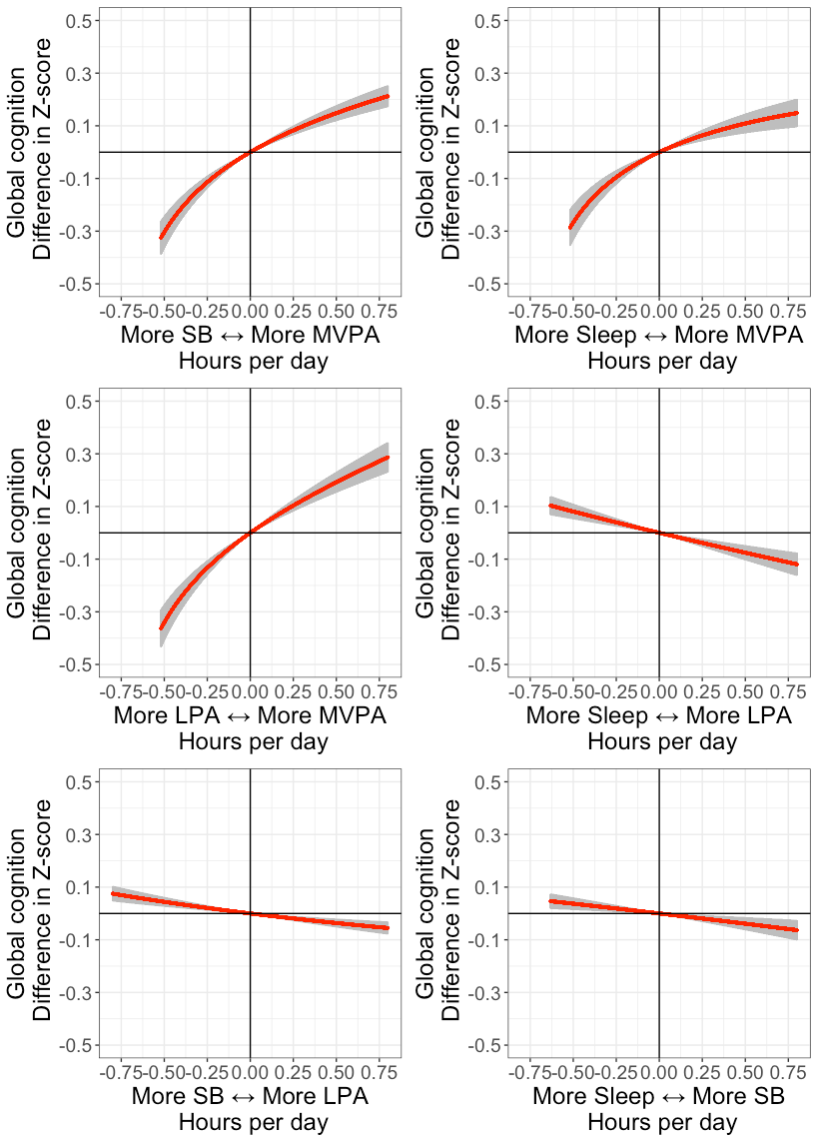


**Supplementary Figure 7.** Predicted age, sex, and education-standardized Z-score of global cognitive function as result of reallocating time between behaviors in participants with insufficient (< 7 hours/day) sleep hours. Models were adjusted for study center, race/ethnicity, body mass index, smoking, excessive alcoholic consumption, and common mental disorders. Compositional references: 6.3 hours/day sleep, 13.5 hours/day SB, 3.5 hours/day LPA, 45min/day MVPA. MVPA: moderate-to-vigorous physical activity. SB: sedentary behavior; LPA: light physical activity.


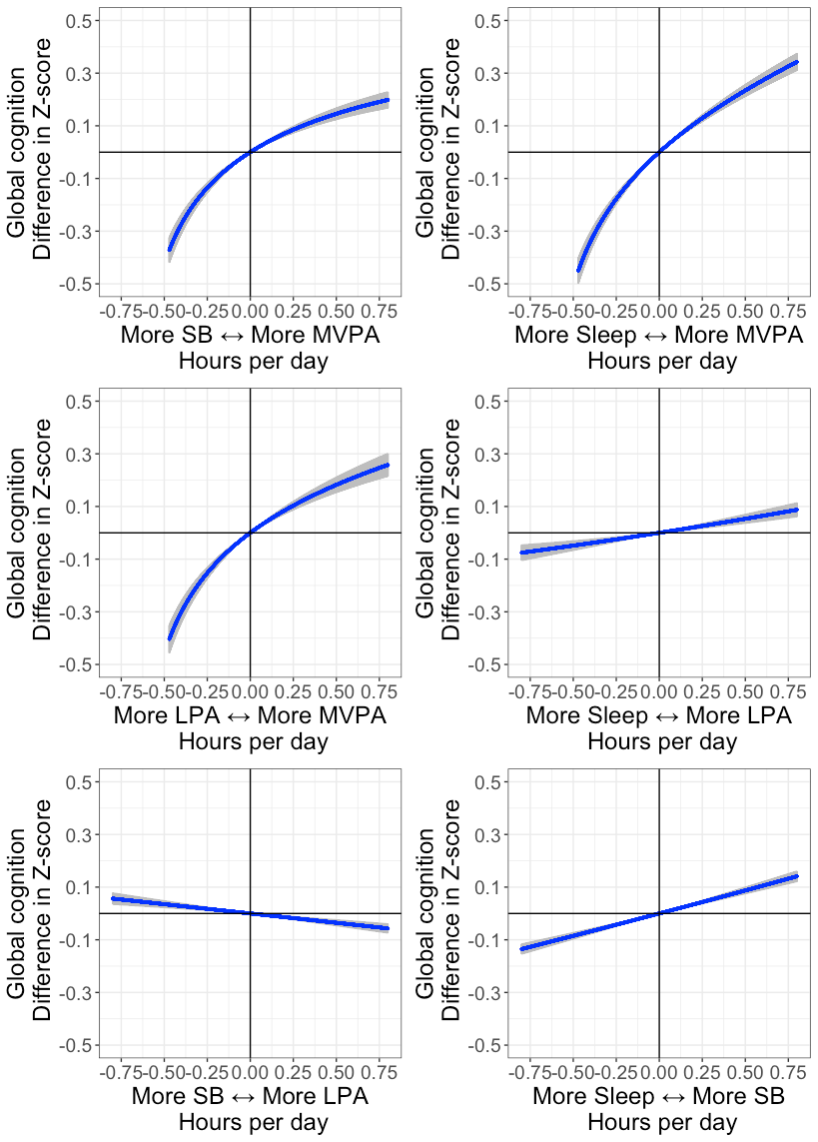


**Supplementary Figure 8.** Predicted age, sex, and education-standardized Z-score of global cognitive function as result of reallocating time between behaviors in participants with sufficient (≥ 7 hours/day) sleep hours. Models were adjusted for study center, race/ethnicity, body mass index, smoking, excessive alcoholic consumption, and common mental disorders. Compositional references: 8.2 hours/day sleep, 11.9 hours/day SB, 3.2 hours/day LPA, 38 min/day MVPA. MVPA: moderate-to-vigorous physical activity. SB: sedentary behavior; LPA: light physical activity.


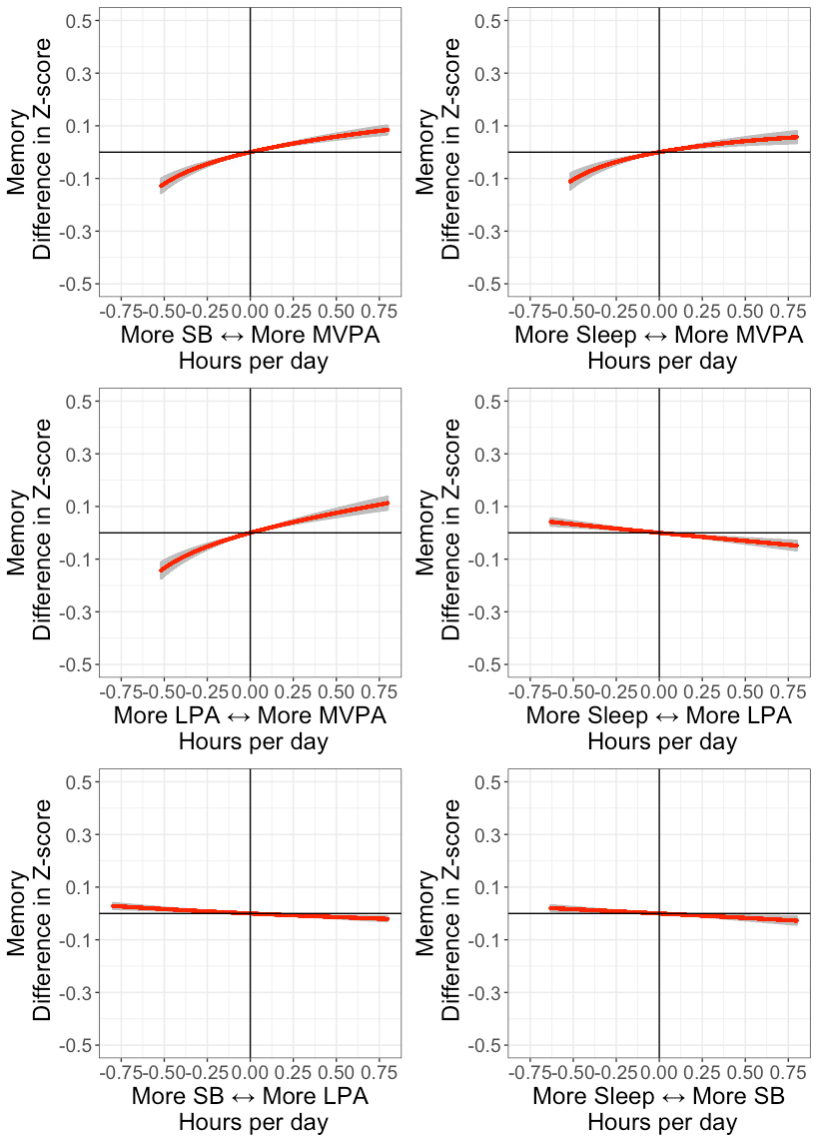


**Supplementary Figure 9.** Predicted age, sex, and education-standardized Z-score of memory function as result of reallocating time between behaviors in participants with insufficient (< 7 hours/day) sleep Models were adjusted for study center, race/ethnicity, body mass index, smoking, excessive alcoholic consumption, and common mental disorders. Compositional references: 6.3 hours/day sleep, 13.5 hours/day SB, 3.5 hours/day LPA, 45min/day MVPA. MVPA: moderate-to-vigorous physical activity. SB: sedentary behavior; LPA: light physical activity.


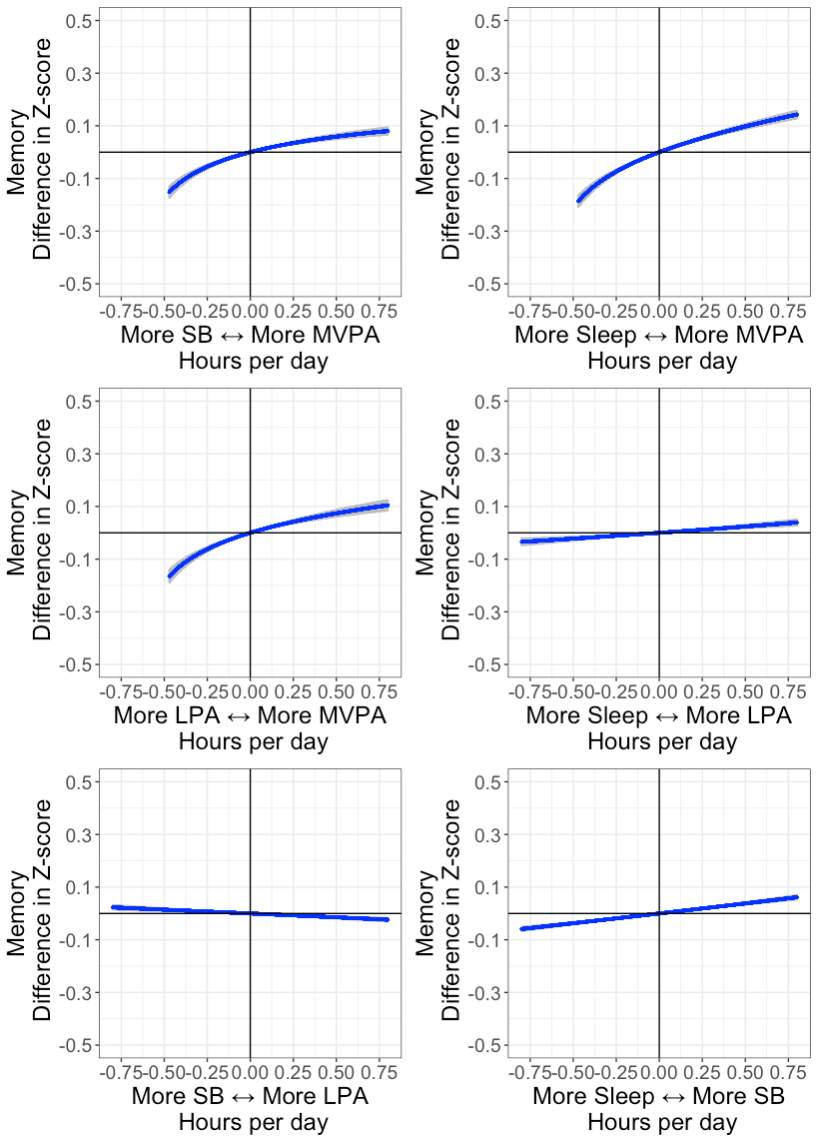


**Supplementary Figure 10.** Predicted age, sex, and education-standardized Z-score of memory function as result of reallocating time between behaviors in participants with sufficient (≥ 7 hours/day) sleep hours. Models were adjusted for study center, race/ethnicity, body mass index, smoking, excessive alcoholic consumption, and common mental disorders. Compositional references: 6.3 hours/day sleep, 13.5 hours/day SB, 3.5 hours/day LPA, 45min/day MVPA. MVPA: moderate-to-vigorous physical activity. SB: sedentary behavior; LPA: light physical activity.


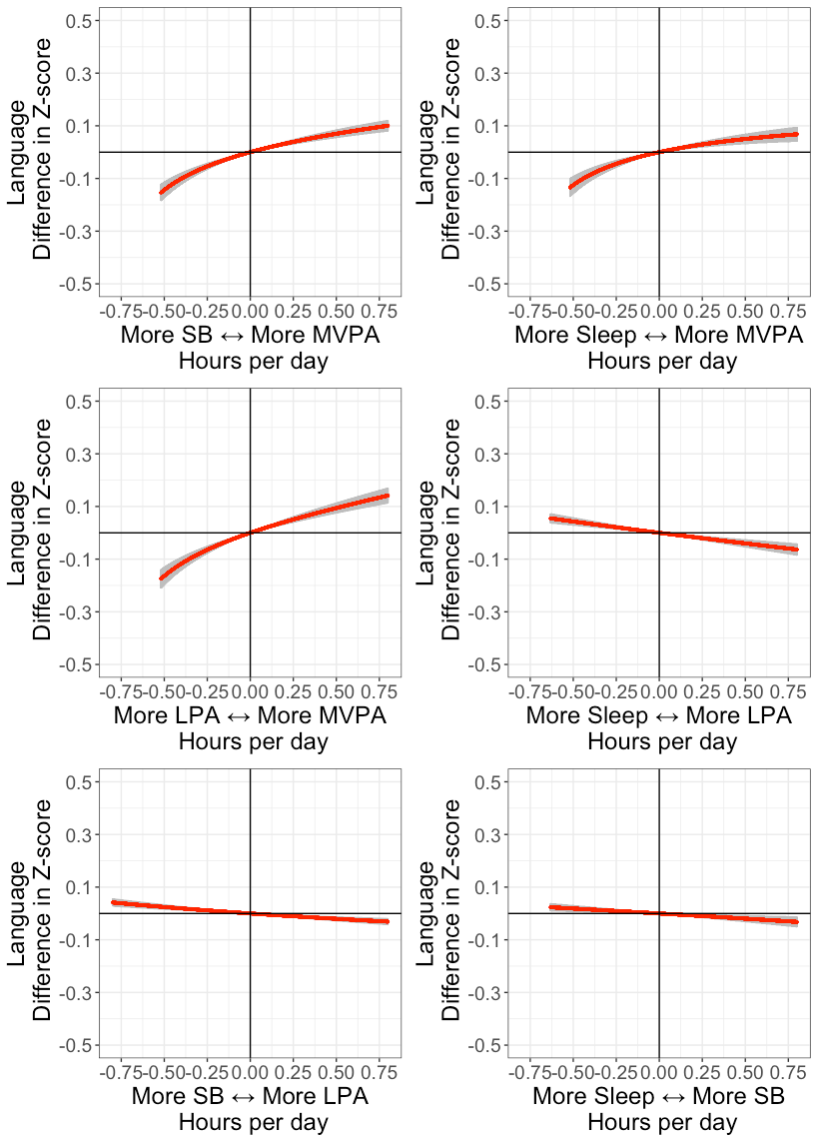


**Supplementary Figure 11.** Predicted age, sex, and education-standardized Z-score of language as result of reallocating time between behaviors in participants with insufficient (< 7 hours/day) sleep hours. Models were adjusted for study center, race/ethnicity, body mass index, smoking, excessive alcoholic consumption, and common mental disorders. Compositional references: 6.3 hours/day sleep, 13.5 hours/day SB, 3.5 hours/day LPA, 45min/day MVPA. MVPA: moderate-to-vigorous physical activity. SB: sedentary behavior; LPA: light physical activity.


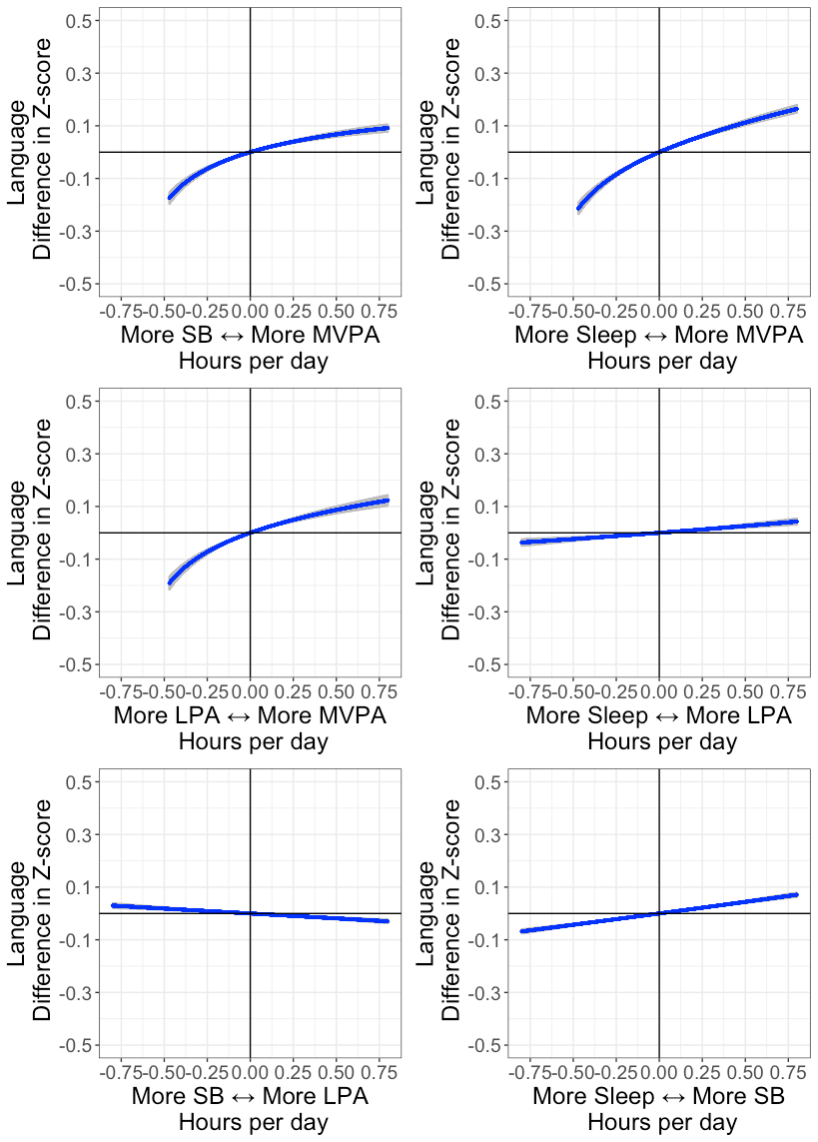


**Supplementary Figure 12.** Predicted age, sex, and education-standardized Z-score of language as result of reallocating time between behaviors in participants with sufficient (≥ 7 hours/day) sleep hours. Models were adjusted for study center, race/ethnicity, body mass index, smoking, excessive alcoholic consumption, and common mental disorders. Compositional references: 8.2 hours/day sleep, 11.9 hours/day SB, 3.2 hours/day LPA, 38 min/day MVPA. MVPA: moderate-to-vigorous physical activity. SB: sedentary behavior; LPA: light physical activity.


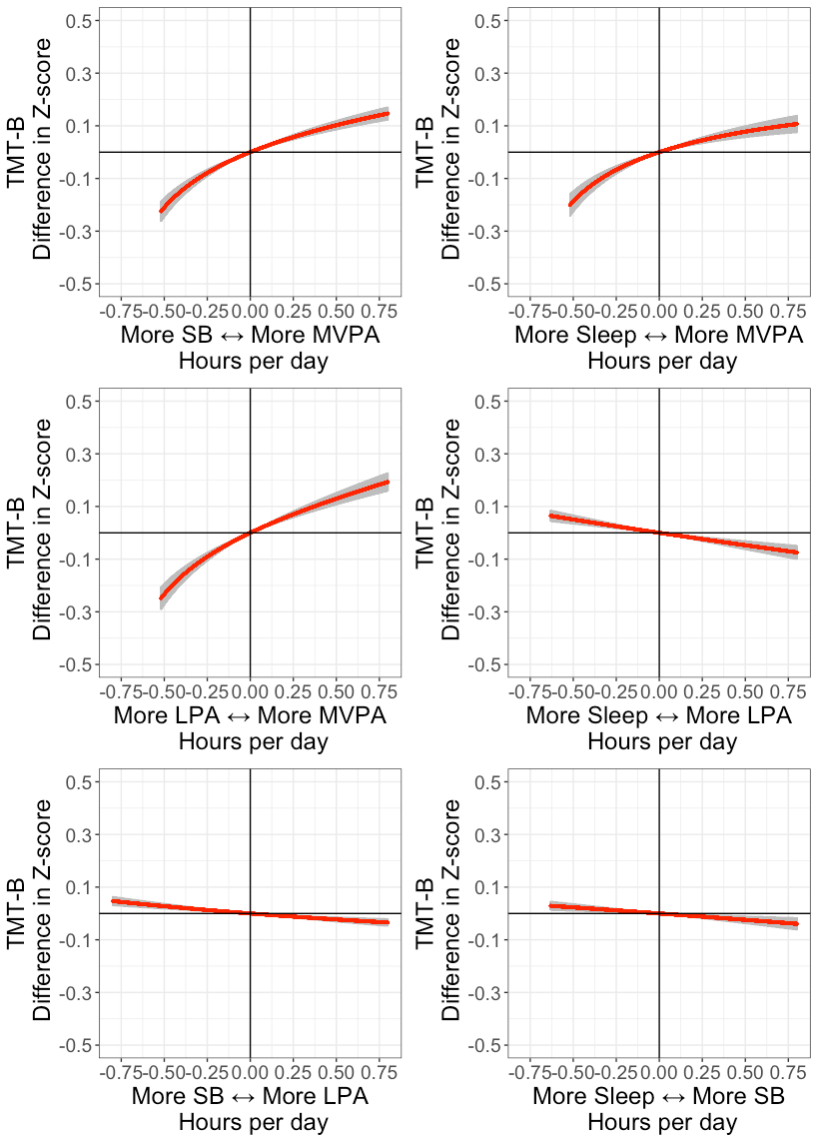


**Supplementary Figure 13.** Predicted age, sex, and education-standardized Z-score of Trail Making test as result of reallocating time between behaviors in participants with insufficient (< 7 hours/day) sleep hours. Models were adjusted for study center, race/ethnicity, body mass index, smoking, excessive alcoholic consumption, and common mental disorders. Compositional references: 6.3 hours/day sleep, 13.5 hours/day SB, 3.5 hours/day LPA, 45min/day MVPA. MVPA: moderate-to-vigorous physical activity. SB: sedentary behavior; LPA: light physical activity.


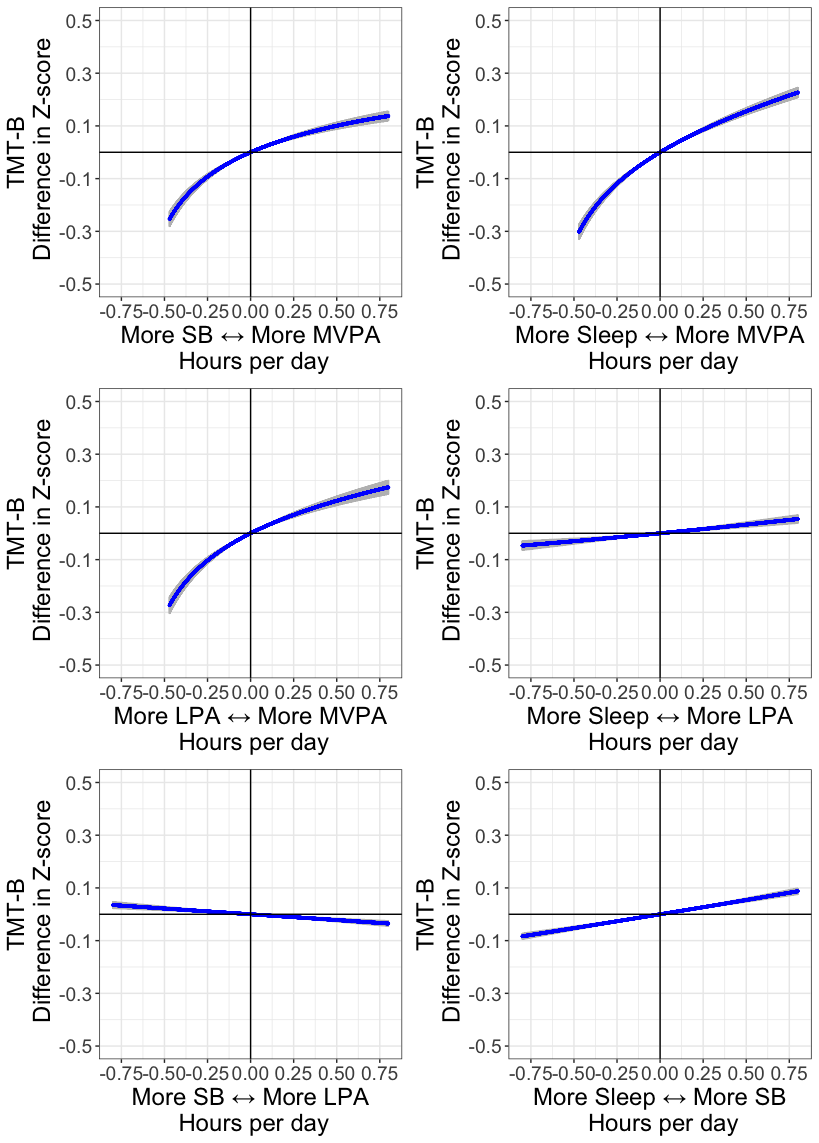


**Supplementary Figure 14.** Predicted age, sex, and education-standardized Z-score of Trail Making test as result of reallocating time between behaviors in participants with sufficient (≥ 7 hours/day) sleep hours. Models were adjusted for study center, race/ethnicity, body mass index, smoking, excessive alcoholic consumption, and common mental disorders. Compositional references: 8.2 hours/day sleep, 11.9 hours/day SB, 3.2 hours/day LPA, 38 min/day MVPA. MVPA: moderate-to-vigorous physical activity. SB: sedentary behavior; LPA: light physical activity.


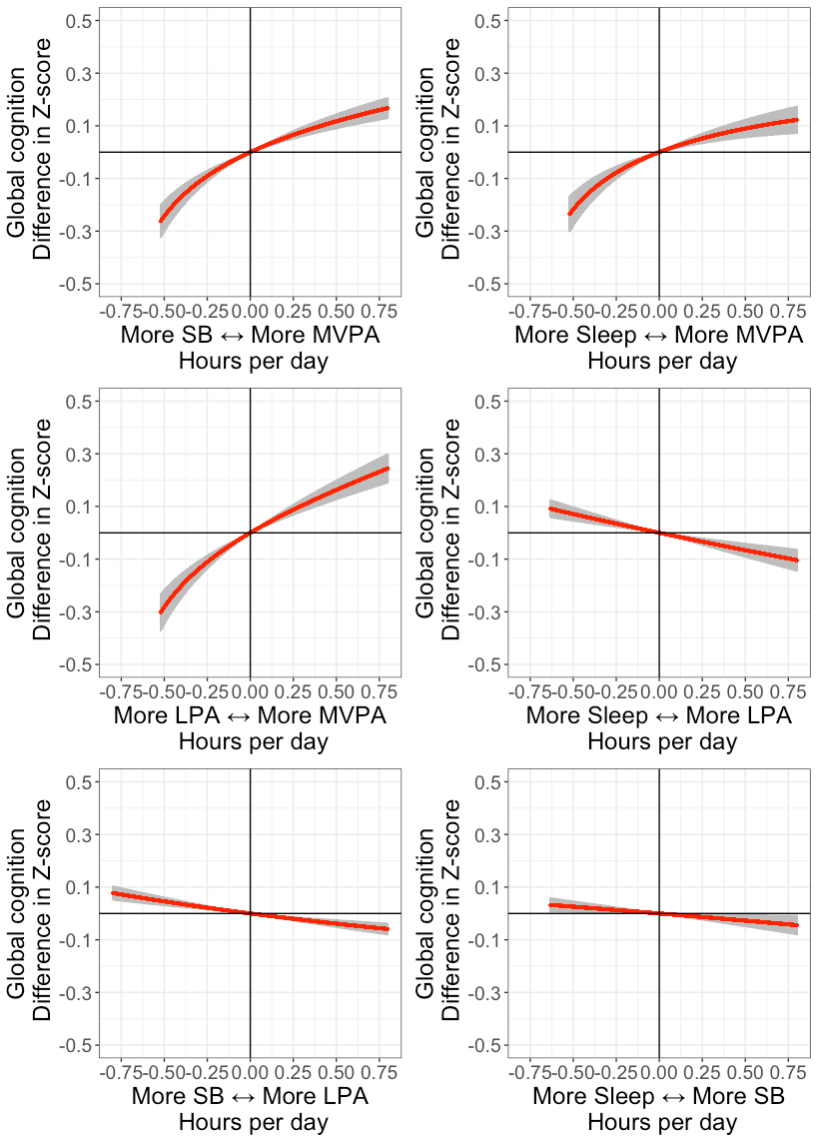


**Supplementary Figure 15.** Sensitivity analysis adjusting for diabetes and hypertension. Predicted age, sex, and education-standardized global cognitive scores as result of reallocating time between behaviors in participants with insufficient (< 7 hours/day) sleep hours. Models were adjusted for study center, race/ethnicity, body mass index, smoking, excessive alcoholic consumption, and common mental disorders. Compositional references: 6.3 hours/day sleep, 13.5 hours/day SB, 3.5 hours/day LPA, 45min/day MVPA. MVPA: moderate-to-vigorous physical activity. SB: sedentary behavior; LPA: light physical activity.


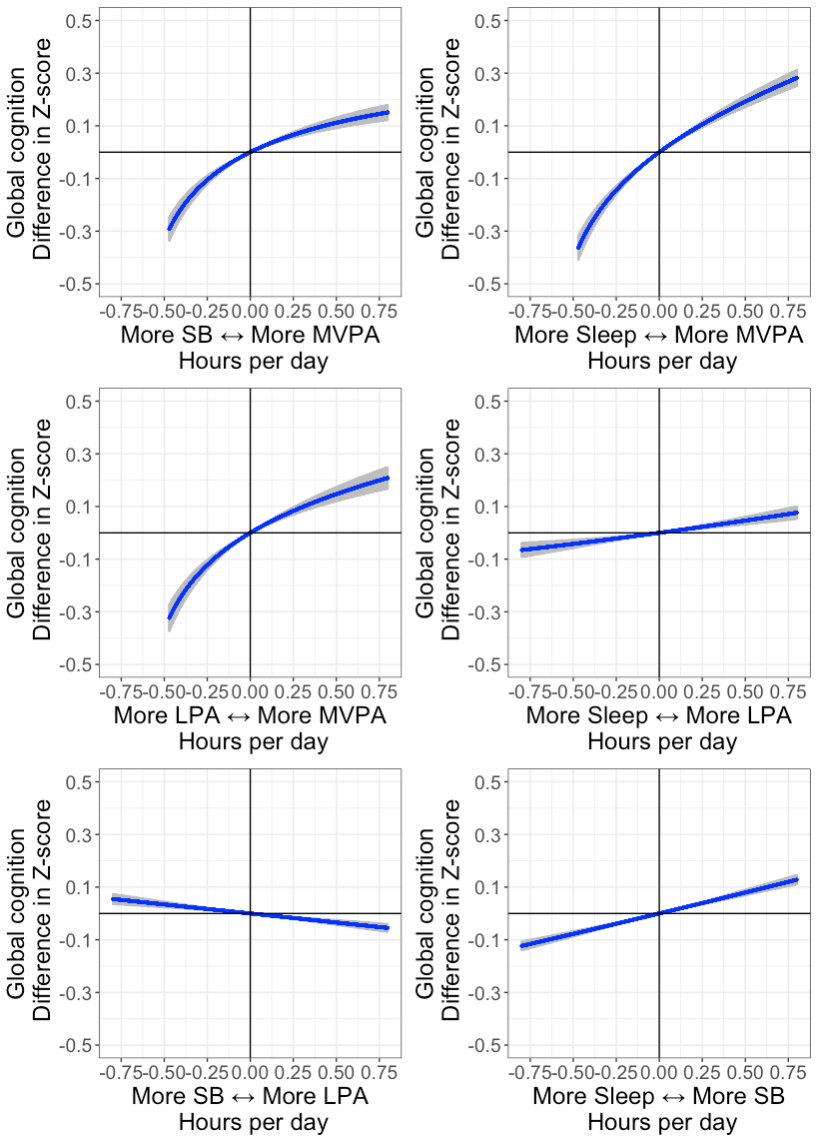


**Supplementary Figure 16.** Sensitivity analysis adjusting for diabetes and hypertension. Predicted age, sex, and education-standardized global cognitive scores as result of reallocating time between behaviors in participants with sufficient (≥ 7 hours/day) sleep hours. Models were adjusted for study center, race/ethnicity, body mass index, smoking, excessive alcoholic consumption, and common mental disorders. Compositional references: 8.2 hours/day sleep, 11.9 hours/day SB, 3.2 hours/day LPA, 38 min/day MVPA. MVPA: moderate-to-vigorous physical activity. SB: sedentary behavior; LPA: light physical activity.


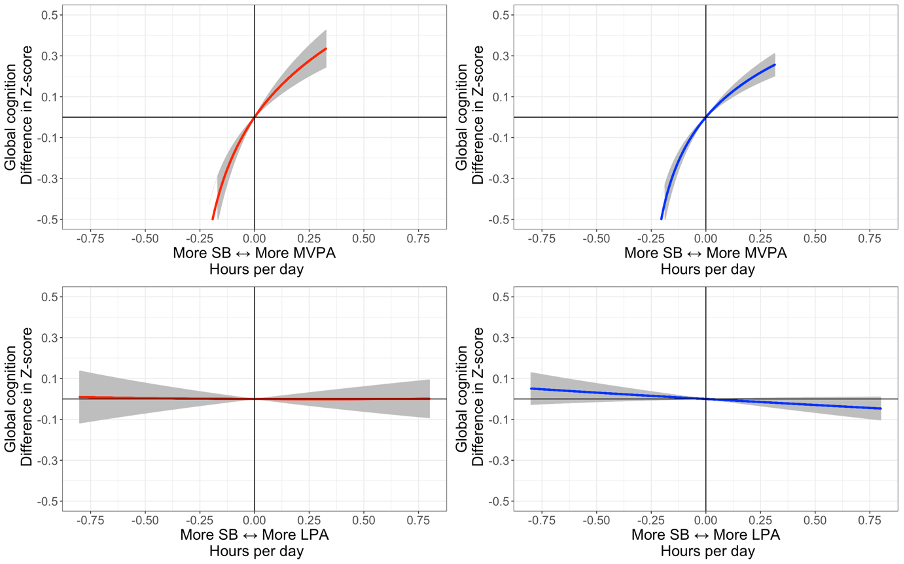


**Supplementary Figure 17.** Sensitivity analysis examining only the lowest (first) decile of mean acceleration per day. Predicted age, sex, and education-standardized global cognition as result of reallocating time between behaviors. Insufficient (< 7 hours/day) and sufficient (≥ 7 hours/day) sleep hours are represented by red and blue lines, respectively. Models were adjusted for study center, race/ethnicity, body mass index, smoking, excessive alcoholic consumption, and common mental disorders. Compositional references: insufficient sleep: 6.2 hours/day sleep, 15.2 hours/day SB, 2.3 hours/day LPA, 16.6 min/day MVPA; sufficient sleep: 8.4 hours/day sleep, 13.1 hours/day SB, 2.3 hours/day LPA, 15.6 min/day MVPA. MVPA: moderate-to-vigorous physical activity. SB: sedentary behavior; LPA: light physical activity.

**Supplementary Table 1.** Arithmetic and compositional mean time spent during the 24-hour movement behavior.

|  | SB (hours/day) | LPA (hours/day) | MVPA (minutes/day) | Sleep (hours/day) |
| --- | --- | --- | --- | --- |
| ***Arithmetic, mean (SD)*** |  |  |  |  |
| Insufficient sleep | 13.3 (1.5) | 3.6 (1.1) | 51.9 (26.3) | 6.0 (0.8) |
| Sufficient sleep | 11.8 (1.5) | 3.3 (1.1) | 45.7 (24.7) | 8.1 (0.9) |
| ***Compositional, mean*** |  |  |  |  |
| Insufficient sleep | 13.5 | 3.5 | 45.2 | 6.3 |
| Sufficient sleep | 11.9 | 3.2 | 38.4 | 8.2 |

LPA: light-intensity physical activity; MVPA: moderate-to-vigorous physical activity; SB: sedentary behavior.

**Supplementary Table 2.** Adjusted^a^ differences in domain-specific cognitive function^b^ estimated to result from minutes of sedentary behavior being reallocated to light (LPA) or moderate-to-vigorous physical activity (MVPA) or greater sleep, the latter stratified by reported current sleep duration. Values are reported as Z-scores and respective 95%CIs.

|  | 15 minutes | 30 minutes | 60 minutes |
| --- | --- | --- | --- |
| ***Sleep < 7 hours/day*** |  |  |  |
| Memory |  |  |  |
| MVPA | 0.03 (0.02, 0.04) | 0.05 (0.04, 0.07) | 0.09 (0.07, 0.11) |
| LPA | -0.01 (-0.01, 0.00) | -0.01 (-0.02, -0.01) | -0.02 (-0.04, -0.01) |
| Sleep | 0.01 (0.00, 0.01) | 0.02 (0.01, 0.03) | 0.03 (0.01, 0.05) |
| Language |  |  |  |
| MVPA | 0.04 (0.03, 0.04) | 0.06 (0.05, 0.08) | 0.11 (0.09, 0.13) |
| LPA | -0.01 (-0.01, -0.01) | -0.02 (-0.03, -0.01) | -0.03 (-0.05, -0.02) |
| Sleep | 0.01 (0.00, 0.01) | 0.02 (0.01, 0.03) | 0.03 (0.01, 0.06) |
| Trail Making test |  |  |  |
| MVPA | 0.05 (0.04, 0.06) | 0.10 (0.08, 0.12) | 0.16 (0.13, 0.19) |
| LPA | -0.01 (-0.02, -0.01) | -0.02 (-0.03, -0.01) | -0.04 (-0.06, -0.02) |
| Sleep | 0.01 (0.00, 0.02) | 0.02 (0.01, 0.03) | 0.04 (0.02, 0.07) |
| ***Sleep ≥ 7 hours/day*** |  |  |  |
| Memory |  |  |  |
| MVPA | 0.03 (0.03, 0.04) | 0.06 (0.05, 0.07) | 0.09 (0.07, 0.11) |
| LPA | -0.01 (-0.01, -0.01) | -0.01 (-0.02, -0.01) | -0.03 (-0.04, -0.02) |
| Sleep | -0.02 (-0.02, -0.02) | -0.04 (-0.04, -0.03) | -0.07 (-0.08, -0.06) |
| Language |  |  |  |
| MVPA | 0.04 (0.03, 0.04) | 0.07 (0.06, 0.07) | 0.10 (0.08, 0.12) |
| LPA | -0.01 (-0.01, -0.01) | -0.02 (-0.03, -0.01) | -0.04 (-0.05, -0.03) |
| Sleep | -0.02 (-0.03, -0.02) | -0.04 (-0.05, -0.04) | -0.08 (-0.09, -0.07) |
| Trail Making test |  |  |  |
| MVPA | 0.05 (0.05, 0.06) | 0.10 (0.08, 0.11) | 0.15 (0.13, 0.17) |
| LPA | -0.01 (-0.02, -0.01) | -0.02 (-0.03, -0.02) | -0.04 (-0.06, -0.03) |
| Sleep | -0.03 (-0.03, -0.02) | -0.05 (-0.06, -0.05) | -0.10 (-0.12, -0.09) |

^a^ Linear regression models adjusted for study center, race/ethnicity, body mass index, smoking, excessive alcohol consumption, and the presence of common mental disorders.

^b^ Estimated difference in age, sex, and education-standardized scores of domain-specific cognitive function.

**Supplementary Table 3.** Adjusted relative difference in the odds of poor cognitive function estimated to result from reallocating minutes of sedentary behavior to light (LPA) or moderate-to-vigorous physical activity (MVPA) or greater sleep, the latter stratified by reported current sleep duration.

|  | 15 minutes  OR (95%CI) | 30 minutes  OR (95%CI) | 60 minutes  OR (95%CI) |
| --- | --- | --- | --- |
| ***Sleep < 7 hours/day*** |  |  |  |
| MVPA | 0.84 (0.80, 0.89) | 0.74 (0.67, 0.82) | 0.60 (0.50, 0.70) |
| LPA | 1.04 (1.00, 1.07) | 1.07 (1.01, 1.14) | 1.14 (1.01, 1.28) |
| Sleep | 0.94 (0.90, 0.97) | 0.88 (0.81, 0.95) | 0.77 (0.67, 0.90) |
| ***Sleep < 7 hours/day*** |  |  |  |
| MVPA | 0.87 (0.83, 0.90) | 0.78 (0.73, 0.84) | 0.69 (0.61, 0.77) |
| LPA | 1.03 (1.01, 1.06) | 1.07 (1.03, 1.12) | 1.15 (1.06, 1.24) |
| Sleep | 1.11 (1.09, 1.13) | 1.22 (1.17, 1.27) | 1.48 (1.37, 1.59) |

Logistic regression models adjusted for study center, race/ethnicity, body mass index, smoking, excessive alcohol consumption, and the presence of common mental disease. Poor cognitive function was defined as age, sex, and education-standardized scores in the first (lowest) decile of the global cognitive function.

**Supplementary Table 4.** Sensitivity analysis on the association of changes in cognitive function, expressed as differences in Z-score, associated with changes in the distribution of 24-hour movement behavior when adjusted additionally for diabetes and hypertension.

|  | 15 minutes  Z-score (95%CI) | 30 minutes  Z-score (95%CI) | 60 minutes  Z-score (95%CI) |
| --- | --- | --- | --- |
| ***Sleep < 7 hours/day*** |  |  |  |
| Global cognition |  |  |  |
| LPA | -0.02 (-0.03, -0.01) | -0.04 (-0.05, -0.02) | -0.07 (-0.10, -0.05) |
| MVPA | 0.07 (0.05, 0.08) | 0.12 (0.09, 0.14) | 0.20 (0.15, 0.24) |
| Sleep | 0.01 (0.00, 0.02) | 0.03 (0.00, 0.05) | 0.05 (0.00, 0.09) |
| Memory |  |  |  |
| LPA | -0.01 (-0.01, -0.00) | -0.01 (-0.02, -0.01) | -0.03 (-0.04, -0.01) |
| MVPA | 0.02 (0.02, 0.03) | 0.04 (0.03, 0.05) | 0.07 (0.05, 0.09) |
| Sleep | 0.01 (0.00, 0.01) | 0.01 (0.00, 0.02) | 0.02 (0.00, 0.04) |
| Language |  |  |  |
| LPA | -0.01 (-0.01, -0.00) | -0.02 (-0.03, -0.01) | -0.04 (-0.05, -0.03) |
| MVPA | 0.03 (0.01, 0.04) | 0.05 (0.04, 0.07) | 0.09 (0.07, 0.12) |
| Sleep | 0.01 (0.00, 0.01) | 0.01 (0.00, 0.02) | 0.03 (0.01, 0.05) |
| Trail Making test |  |  |  |
| LPA | -0.02 (-0.01, -0.01) | -0.02 (-0.03, -0.02) | -0.05 (-0.06, -0.03) |
| MVPA | 0.05 (0.04, 0.06) | 0.08 (0.07, 0.10) | 0.14 (0.11, 0.17) |
| Sleep | 0.01 (0.00, 0.02) | 0.02 (0.00, 0.03) | 0.03 (0.01, 0.06) |
| ***Sleep < 7 hours/day*** |  |  |  |
| Global cognition |  |  |  |
| LPA | -0.04 (-0.05, -0.03) | -0.04 (-0.05, -0.03) | -0.07 (-0.09, -0.05) |
| MVPA | 0.07 (0.05, 0.08) | 0.11 (0.09, 0.13) | 0.21 (0.15, 0.27) |
| Sleep | 0.00 (-0.00, 0.01) | -0.08 (-0.09, -0.07) | -0.15 (-0.18, -0.13) |
| Memory |  |  |  |
| LPA | -0.01 (-0.01, -0.00) | -0.01 (-0.02, -0.01) | -0.03 (-0.04, -0.02) |
| MVPA | 0.02 (0.02, 0.03) | 0.04 (0.03, 0.05) | 0.06 (0.05, 0.08) |
| Sleep | -0.02 (-0.02, -0.01) | -0.03 (-0.04, -0.03) | -0.07 (-0.08, -0.06) |
| Language |  |  |  |
| LPA | -0.01 (-0.01, -0.01) | -0.02 (-0.02, -0.01) | -0.04 (-0.05, -0.03) |
| MVPA | 0.03 (0.02, 0.04) | 0.05 (0.04, 0.06) | 0.08 (0.07, 0.10) |
| Sleep | -0.02 (-0.02, -0.02) | -0.04 (-0.05, -0.03) | -0.08 (-0.09, -0.07) |
| Trail Making test |  |  |  |
| LPA | -0.01 (-0.01, -0.00) | -0.02 (-0.03, -0.02) | -0.04 (-0.06, -0.03) |
| MVPA | 0.05 (0.04, 0.05) | 0.08 (0.07, 0.09) | 0.13 (0.10, 0.15) |
| Sleep | -0.03 (-0.03, -0.02) | -0.05 (-0.05, -0.04) | -0.10 (-0.11, -0.08) |

Estimated difference in age, sex, and education-standardized scores of domain-specific cognitive function. Multiple linear regression models adjusted for study center, race/ethnicity, body mass index, smoking, excessive alcoholic consumption, common mental disorder, diabetes, and hypertension.

**Supplementary Table 5.** Sensitivity analysis on the association of changes in cognitive function, expressed as differences in odds of low (1^st^ decile) function, associated with changes in the distribution of 24-hour movement behavior when adjusted additionally for diabetes and hypertension.

|  | 15 minutes  OR (95%CI) | 30 minutes  OR (95%CI) | 60 minutes  OR (95%CI) |
| --- | --- | --- | --- |
| ***Sleep < 7 hours/day*** |  |  |  |
| LPA | 1.04 (1.01, 1.08) | 1.02 (1.02, 1.15) | 1.15 (1.02, 1.30) |
| MVPA | 0.87 (0.82, 0.92) | 0.78 (0.71, 0.86) | 0.66 (0.55, 0.78) |
| Sleep | 0.94 (0.91, 0.98) | 0.89 (0.82, 0.96) | 0.80 (0.68, 0.93) |
| ***Sleep < 7 hours/day*** |  |  |  |
| LPA | 1.03 (1.01, 1.06) | 1.07 (1.03, 1.11) | 1.14 (1.06, 1.23) |
| MVPA | 0.90 (0.86, 0.93) | 0.83 (0.77, 0.89) | 0.76 (0.67, 0.86) |
| Sleep | 1.10 (1.08, 1.12) | 1.21 (1.16, 1.25) | 1.45 (1.34, 1.56) |

Logistic regression models adjusted for study center, race/ethnicity, body mass index, smoking, excessive alcohol consumption, presence of common mental disease, diabetes, and hypertension. Poor cognitive function was defined as age, sex, and education-standardized scores in the first (lowest) decile of the global cognitive function.

**Supplementary Table 6.** Arithmetical and compositional mean time spent during the 24-hour movement behavior in the lowest decile of mean daily acceleration.

|  | SB (hours/day) | LPA (hours/day) | MVPA (minutes/day) | Sleep (hours/day) |
| --- | --- | --- | --- | --- |
| ***Arithmetic, mean and (SD)*** |  |  |  |  |
| Insufficient sleep | 15.1 (0.9) | 2.4 (0.6) | 19.8 (9.7) | 6.2 (0.7) |
| Sufficient sleep | 13.0 (1.2) | 2.3 (0.6) | 19.0 (9.6) | 8.4 (1.0) |
| ***Compositional, mean*** |  |  |  |  |
| Insufficient sleep | 15.2 | 2.3 | 16.6 | 6.2 |
| Sufficient sleep | 13.1 | 2.3 | 15.6 | 8.4 |

LPA: light-intensity physical activity; MVPA: moderate-to-vigorous physical activity; SB: sedentary behavior.

**Supplementary Table 7.** Characteristics of participants according to availability of accelerometer data in wave 3 of the ELSA-Brasil study. N=12,636

| Characteristic | No missing data (n=9,354) | | | Missing data (n=3,282) | | | p-value |
| --- | --- | --- | --- | --- | --- | --- | --- |
|  | Mean (SD) | N (%) | Median (IQR) | Mean (SD) | N (%) | Median (IQR) |  |
| Age, years | 51.09 (8.68) |  |  | 52.34 (9.32) |  |  | <0.001 |
| Sex |  |  |  |  |  |  | 0.820 |
| Male | 4,157 (44.4%) |  |  | 1,466 (44.7%) |  |  |  |
| Female | 5,197 (55.6%) |  |  | 1,816 (55.3%) |  |  |  |
| Race or race/ethnicity |  |  |  |  |  |  | 0.002 |
| Black |  | 1,418 (15.3%) |  |  | 566 (17.5%) |  |  |
| Mixed race (*pardo)* |  | 2,543 (27.4%) |  |  | 917 (28.4%) |  |  |
| White |  | 4,987 (53.8%) |  |  | 1,624 (50.2%) |  |  |
| Asian or Indigenous |  | 319 (3.4%) |  |  | 125 (3.9%) |  |  |
| Highest educational achievement |  |  |  |  |  |  | <0.001 |
| Less than elementary school |  | 383 (4.1%) |  |  | 200 (6.1%) |  |  |
| Elementary school |  | 515 (5.5%) |  |  | 246 (7.5%) |  |  |
| High school |  | 3,243 (34.7%) |  |  | 1,120 (34.1%) |  |  |
| University degree or higher |  | 5,213 (55.7%) |  |  | 1,716 (52.3%) |  |  |
| Smoking |  |  |  |  |  |  | <0.001 |
| Never smoker |  | 5,558 (59.4%) |  |  | 1,849 (56.3%) |  |  |
| Former smoker |  | 2,741 (29.3%) |  |  | 974 (29.7%) |  |  |
| Current smoker |  | 1,055 (11.3%) |  |  | 459 (14.0%) |  |  |
| Excessive alcohol drinking, yes ^a^ |  | 564 (6.0%) |  |  | 183 (5.6%) |  | 0.350 |
| Sleep, hours per night |  |  |  |  |  |  | <0.001 |
| <7 |  | 3,284 (35.4%) |  |  | 1,578 (49.9%) |  |  |
| ≥7 |  | 5,986 (64.6%) |  |  | 1,583 (50.1%) |  |  |
| Body Mass Index |  |  |  |  |  |  | 0.170 |
| Overweight |  | 3,452 (36.9%) |  |  | 1,185 (36.1%) |  |  |
| Obese |  | 3,765 (40.3%) |  |  | 1,305 (39.8%) |  |  |
| Diabetes, yes |  | 1,301 (13.9%) |  |  | 518 (15.8%) |  | 0.009 |
| Hypertension, yes (n and %) |  | 3,062 (32.8%) |  |  | 1,188 (36.2%) |  | <0.001 |
| Common mental disorder, yes ^b^ |  | 2,447 (26.2%) |  |  | 917 (28.0%) |  | 0.046 |
| Memory, number words recalled ^c^ | 38.19 (5.72) |  |  | 37.31 (6.08) |  |  | <0.001 |
| Language, number words recalled ^d^ | 31.67 (8.07) |  |  | 30.52 (8.58) |  |  | <0.001 |
| Trail Making Test (part B), seconds ^e^ |  |  | 93.00 (72.00, 131.00) |  |  | 101.00 (75.00, 145.00) | <0.001 |

^a^ ≥210 g of alcohol/week for men and ≥140 g of alcohol/week for women.

^b^ Score ≥12 in the *Clinical Interview Schedule-Revised* (CIS-R).

^c^ Range from 0 to 50.

^d^ Range from 0 to ∞

^e^ Time to complete the task

^f^ Acceleration ≤15m*g*

^g^ Acceleration 16-69m*g*

^h^ Acceleration >69m*g*.

^i^ Reported in sleep diary.
